# The structural basis of fungal glucuronoyl esterase activity on natural substrates

**DOI:** 10.1038/s41467-020-14833-9

**Published:** 2020-02-24

**Authors:** Heidi A. Ernst, Caroline Mosbech, Annette E. Langkilde, Peter Westh, Anne S. Meyer, Jane W. Agger, Sine Larsen

**Affiliations:** 1 Department of Chemistry, University of Copenhagen, Universitetsparken 5, 2100 Copenhagen Ø, Denmark; 2 Department of Biotechnology and Biomedicine, Technical University of Denmark, Søltofts Plads 221, 2800 Kongens Lyngby, Denmark; 3 Department of Drug Design and Pharmacology, University of Copenhagen, Universitetsparken 2, 2100 Copenhagen Ø, Denmark

**Keywords:** Biocatalysis, Hydrolases

## Abstract

Structural and functional studies were conducted of the glucuronoyl esterase (GE) from *Cerrena unicolor* (*Cu*GE), an enzyme catalyzing cleavage of lignin-carbohydrate ester bonds. *Cu*GE is an α/β-hydrolase belonging to carbohydrate esterase family 15 (CE15). The enzyme is modular, comprised of a catalytic and a carbohydrate-binding domain. SAXS data show *Cu*GE as an elongated rigid molecule where the two domains are connected by a rigid linker. Detailed structural information of the catalytic domain in its apo- and inactivated form and complexes with aldouronic acids reveal well-defined binding of the 4-*O*-methyl-a-D-glucuronoyl moiety, not influenced by the nature of the attached xylo-oligosaccharide. Structural and sequence comparisons within CE15 enzymes reveal two distinct structural subgroups. *Cu*GE belongs to the group of fungal CE15-B enzymes with an open and flat substrate-binding site. The interactions between *Cu*GE and its natural substrates are explained and rationalized by the structural results, microscale thermophoresis and isothermal calorimetry.

## Abstract (web summary)

Glucuronoyl esterases have the potential to be used in the biocatalytic conversion of lignin-carbohydrate complexes to obtain pure lignin for downstream biofuel conversion. Here the authors present a detailed structural analysis of the glucuronoyl esterase from *Cerrena unicolor*, providing the basis for its activity on natural substrate and for how lignin can be selectively separated from lignocellulosic materials.

## Introduction

**Fig. 1:**
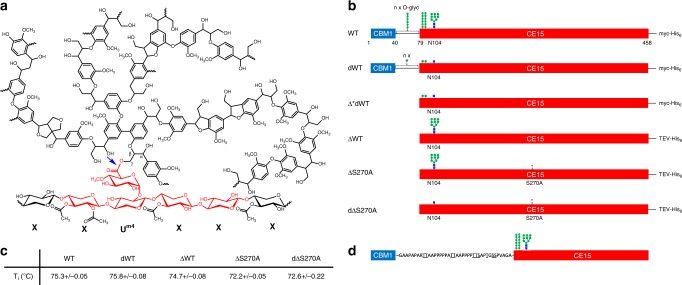
Proposed natural LCC substrate and *Cu*GE variants. **a** Structural representation of a natural LCC substrate for glucuronoyl esterases. The ester-linked LCC (blue arrow) connects two polymeric structures; acetylated glucuronoxylan substituted with α-1,2-linked 4-*O*-methyl-glucuronoyl moieties and lignin. The 4-*O*-methyl-glucuronoyls form ester bonds with γ-alcohols from lignin. The carbohydrate structure corresponding to the three different ligands in the *Cu*GE crystallographic complexes are marked in red. Each xylosyl unit is named X and the 4-*O*-methyl-glucuronoyl substituted xylosyl is named U^m4^. **b** *Cu*GE variants used in this study. The full-length enzyme (WT) has a modular structure with a CBM1 domain appended to the catalytic CE15 domain via a linker. Positions that are post translationally modified in the recombinant enzyme with either N- or O-glycosylation are stylized (NAG blue squares; MAN green dots). Here, only the core structure of the glycans is shown, but especially the N-glycosylation is quite extensive and heterogeneous in the recombinant enzyme produced in *Pichia pastoris*. “Δ” symbolizes a truncated variant without the CBM1 and linker region. In the inactive ΔS270A variant the catalytic nucleophile S270 has been changed to an alanine. “d” denotes variants that have been enzymatically deglycosylated. Notably, the cleaved variant Δ*dWT was spontaneously produced in crystallization experiments with dWT. **c** Relative stability of the *Cu*GE variants. *T*_i_ is the apparent thermal unfolding temperature derived from changes in intrinsic fluorescence and stated with standard deviations. **d** Sequence of the proline-rich linker region in *Cu*GE. Potential O-glycosylation sites (S or T) are underlined. Source data for panel **c** are provided as a Source Data file.

Lignin-carbohydrate complexes (LCCs) play an integral role in maintaining plant cell wall integrity and recalcitrance of lignocellulosic biomass^1,2^. LCCs represent a major obstacle for the complete utilization of all plant cell wall components, which is a cornerstone in the future bioeconomy-based society because lignin is an important natural resource for replacing fossil-based products and materials with sustainable alternatives^3^. To fully exploit the potential of lignin, it is essential to recover lignin in a non-destructive manner and in high purity, free from residual carbohydrates. A common type of LCC in hardwood is the ester-LCC formed between α-1,2-linked 4-*O*-methyl-d-glucuronoyl moieties of acetylated glucuronoxylan and γ-alcohols in lignin^4^ (Fig. 1a). It was recently discovered that an insoluble hardwood lignin-preparation (LRP) is amenable to enzymatic hydrolysis by a glucuronoyl esterase (GE, EC 3.1.1.B11) releasing aldouronic acids^5^. This opens for the opportunity to use glucuronoyl esterases as a benign tool for processing of lignin in selective removal of residual carbohydrates in contrast to the harsh physio/chemical extractions otherwise used.

GEs are widespread in nature and found in fungal and bacterial lignocellulose degrading organisms^6,7^. The currently known GEs belong to the CE15 family^8^ in the CAZy database. The first crystal structure of a CE15 GE, Cip2 from *Hypocrea jecorina*^9^ and site-directed mutagenesis studies^10^ established that the CE15 enzymes belong to the α/β-hydrolase superfamily with a classical catalytic nucleophile-acid-base (serine-glutamate-histidine) triad and their catalytic function conforms to other enzymes possessing an equivalent catalytic machinery^11^.

The CE15 family is diverse and structural studies of fungal^9,12^ and bacterial^13–15^ members have revealed significant differences within the family, including variations in catalytic machinery and active site topology. There is only one report of a CE15 complex, the *St*GE2:monosaccharide complex^12^, unfortunately this study is not supported by any functional data.

Studies on substrate analogs suggest that the 4-*O*-methylation on the glucuronoyl substitutions in xylan is important for substrate recognition^16,17^. While the ester linkage of the LCC itself may seem well-defined in terms of acid and alcohol donors (Fig. 1a), the structural variations of its surroundings, such as the degree and position of acetylations on the xylan backbone, the amount of glucuronoyl substitutions, the distance between two or more ester linkages, the composition of lignin and the average molecular size of both the carbohydrate and the lignin moieties add complexity and heterogeneity to LCCs as substrate. We have shown previously, that a two-domain glucuronoyl esterase from the basidiomycete *Cerrena unicolor* (*Cu*GE) with high efficiency can catalyze the release of large soluble aldouronic acids from a lignin-rich birchwood fraction, most likely containing the already mentioned ester-linked LCCs^5^.

In the following, our results from extensive structural and functional studies of *Cu*GE are presented. This includes structural investigations by small-angle X-ray scattering (SAXS) of the full-length enzyme, crystal structures of the apo form of the catalytic domain, as well as four relevant complexes with aldouronic acids, and binding affinity measurements on natural insoluble lignin substrate, containing LCCs and soluble substrate analogs. Collectively, these results give us important structural and functional knowledge clarifying the interactions between *Cu*GE and the α-1,2-linked 4-*O*-methyl-d-glucuronoyl moieties on xylo-oligomers and explaining how the enzyme accomodates large complex substrates. Furthermore, a comparative study with other members of the CE15 family reveals two distinct groups of enzymes, A and B of which *Cu*GE belongs to group B. The two groups differ in the position of their catalytic acid and topology, which indicate differences in substrate binding. This overall knowledge on the CE15 enzymes will pave the ground for the understanding of how lignin can be selectively separated from the lignocellulosic cell wall via enzymatic cleavage of ester-linked LCCs.

## Results

### *Cu*GE variants

WT *Cu*GE has a modular architecture with a N-terminal carbohydrate-binding module 1 (CBM1) domain^17,18^ connected to the catalytic domain by a proline-rich linker region. The structure of *Cu*GE is potentially flexible and together with heterogenous N- and O-glycosylation^17^ it could represent an obstacle for high-resolution structural studies of the full-length enzyme (WT). To enable structural and functional studies two different variants were constructed, ΔWT, comprises residues 78–458 lacking the carbohydrate-binding domain (CBM1), and ΔS270A an inactive variant of ΔWT where the catalytic serine is replaced with an alanine (Fig. 1b–d). These *Cu*GE variants were recombinantly produced in *Pichia pastoris* and subsequently purified. For the structural studies, WT and ΔS270A were enzymatically deglycosylated to obtain homogeneous samples, named dWT and dΔS270A, respectively (Fig. 1b-c). Comparative thermal unfolding measurements on the preparations show that neither the truncation nor the deglycosylation affects protein stability significantly (Fig. 1c). The inactive variant ΔS270A is destabilized by ca 3 °C compared to variants with an intact catalytic triad, implying that some stabilizing interactions have been lost, but the overall conclusion is that *Cu*GE is a highly stable enzyme with *T*_i_’s in the range of 72.2–75.8 °C for all variants. Previous studies have shown that both WT and ΔWT *Cu*GE are fully active on both soluble and insoluble substrates^18^.

### Overall structure of *Cu*GE by small-angle X-ray scattering

**Fig. 2:**
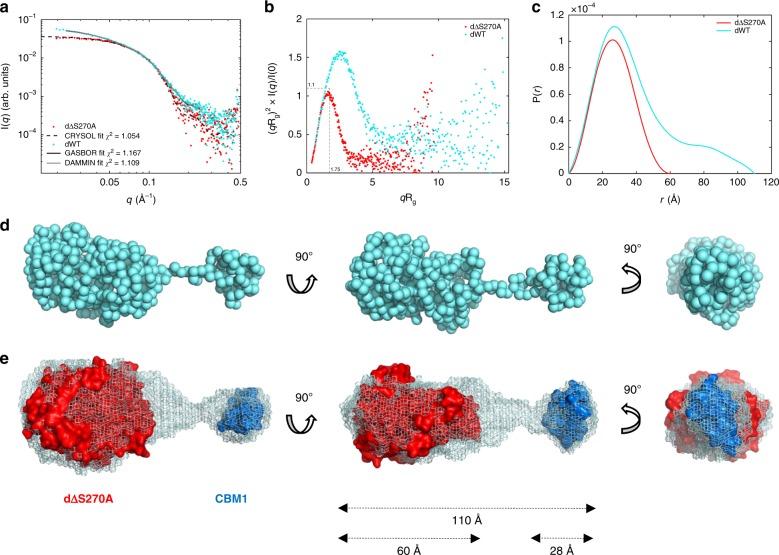
SAXS analyses and models of full-length *Cu*GE. **a** SAXS scattering profiles for truncated, dΔS270A (3.6 mg mL^−1^), and full-length, dWT (3.7 mg mL^−1^), *Cu*GE shown on double logarithmic scales. The CRYSOL fit shows an excellent agreement between the solution structure of dΔS270A and the crystal structure. For dWT, the fits obtained by ab-initio methods, here GASBOR and DAMMIN, respectively, are depicted (black and gray lines, almost fully overlapping). **b** Dimensionless Kratky plots indicating folded and rigid structures. **c** Pair distance distribution functions, P(*r*), with a *D*_max_ of 60 Å for dΔS270A and 110 Å for dWT, respectively. **d** Ab-initio GASBOR model of dWT. **e** Ab-initio DAMMIN model of dWT with the crystal structure of the catalytic domain and a *Cu*GE-CBM1 homology model (Supplementary Fig. 2) placed in the molecular envelope.

In-house small-angle X-ray scattering measurements were performed on dWT and dΔS270A (Fig. 2a). No concentration dependence was observed in the investigated concentration range (Supplementary Fig. 1), and the estimated molecular weights correspond well to monomeric species for both variants. Furthermore, the dimensionless Kratky plots shown in Fig. 2b for both samples, with their distinct bell-shaped curves reaching a plateau near zero at higher *q*-values, display the characteristics of rigid folded proteins. With a peak position in the dimensionless Kratky plot at (sR_g_)^2^ = 1.1, the data for the dΔS270A variant indicate a compact and globular structure. The maximum interatomic distance (*D*_max_) is 60 Å, derived from the pair distribution function P(*r*) (Fig. 2c).

The SAXS data of the full-length protein dWT, with an increased and right-shifted peak position in the dimensionless Kratky plot, display the characteristics of a multi domain elongated yet rigid particle. The pair distance distribution (Fig. 2c) includes both the profile of the catalytic domain and a second peak with relatively constant interdomain distance consistent with the presence of the CBM1 and a *D*_max_ of 110 Å. Ab-initio models of dWT (Fig. 2d, e) reveal a relatively extended structure formed of two domains connected by the 40-residue long linker. The structure of full-length *Cu*GE thus conforms to the emerging picture that linker regions in many CAZymes can be quite extended and rigid^19–22^. These properties appear to be associated with the high content of proline, threonine and serine in the linker region (Fig. 1d and Supplementary Material Fig. 2), which provides stiffness and potentially heavy O-glycosylation that may further reduce the flexibility of the linker^19^. Unfortunately it was not possible to unambiguously determine the conformation of the linker from the SAXS data.

The high-resolution structure of the catalytic domain (dΔS270A) obtained from the crystal structure determinations (described in detail later) matched the solution structure (Fig. 2a). This crystal structure and a homology model of *Cu*GE-CBM1 (Supplementary Fig. 2) were placed in the refined DAMMIN envelope for reference (Fig. 2e). Owing to the globular nature of the individual domains the relative orientation of the two domains remains ambiguous.

### Structure of the catalytic domain of *Cu*GE

**Fig. 3:**
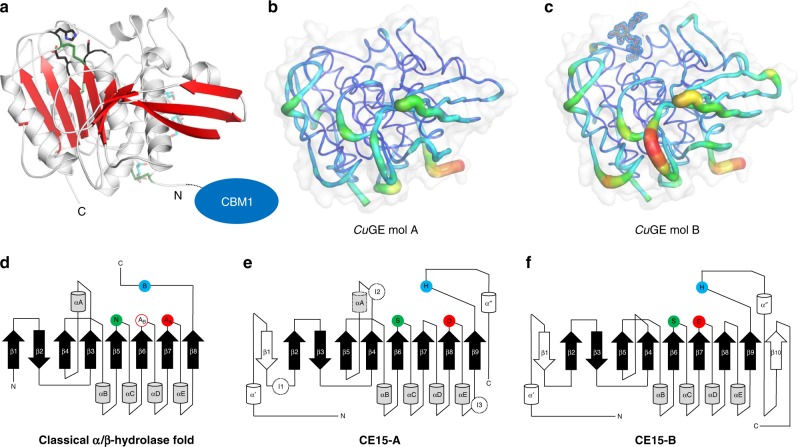
Overall *Cu*GE structure, CE15 subgroups, and relation to the classical α/β-hydrolase fold. **a** Structure of the catalytic domain of *Cu*GE (residues 79–458) with selected residues shown as sticks: the catalytic triad (S270, E293, and H404, black), disulfide bridges (C81–C116, C269–C405, C391–C377, green), and carbohydrates (cyan), indicating the location of N- and O-glycosylation sites (N104-NAG, S86-MAN, and T87-MAN). The break in strand β3 is caused by a proline residue (P169) in *Cu*GE. **b**, **c** The two crystallographically independent molecules A and B from the dΔS270A:U^m4^X complex colored by temperature factors to visualize flexibility. **b** Molecule A. **c** Ligand-bound molecule B. **d** Topology diagram for a classical α/β-hydrolase fold. The illustration is based on information in Ollis et al.^23^, Carr et al.^24^, and Dimitrou et al.^25^. The location of the catalytic residues is shown (nucleophile serine in green, base histidine in blue, and acid glutamate in red). The α/β-hydrolase group A and B are distinguished by different configurations of the catalytic triad with the catalytic acid located either in the turn after β7 (A_A_) or after β6 (A_B_), respectively^25^. **e** Topology diagram for CE15-A. I1-I3 indicate inserts observed in bacterial CE15-A structures^13–15^. **f** Topology diagram for CE15-B. For clarity, only the major secondary structure elements have been included in the topology diagrams in **e**, **f**; structures from both CE15 subgroups comprise additional small secondary structure elements, including multiple helical elements inserted after the strands β5 and β7 (Supplementary Fig. 4).

**Fig. 4:**
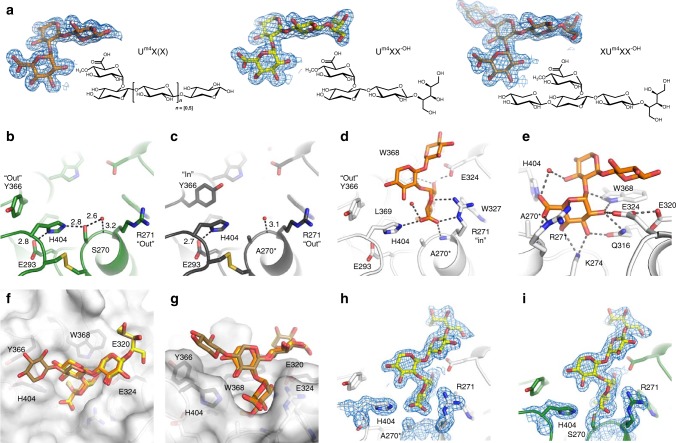
Active site, crystallographic complexes, and XU^m4^X-recognition. **a** Aldouronic acid ligands in the complexes dΔS270A:U^m4^X, dΔS270A:U^m4^XX^-OH^, and dΔS270A:XU^m4^XX^-OH^. 2F_o_–F_c_ electron density maps for the refined ligands are shown at the 1.0 σ level. **b** Apo-Δ*dWT with an intact active site (pH 5.5). Hydrogen bonds between residues in the catalytic triad are indicated. **c** Apo-dΔS270A (pH ca. 7.5) with Y366 in a different conformation (molecule B only). Loss of interactions to S270 might explain the slight destabilization observed for the inactive variant (Fig. 1d). **d** Ligand-bound structure, dΔS270A:U^m4^X(X) (pH ca. 7.5), shown in the same orientation as in **b**, **c** for a direct comparison with the unliganded structures. Note that two movements are observed upon substrate binding: Y366 moves “out”, whereas R271 moves “in” and participates in substrate recognition and formation of the oxyanion hole. **e** dΔS270A:U^m4^X(X) complex shown in a different perspective to visualize the extensive network of hydrogen bonds in recognition of the 4-*O*-methyl-α-d-glucuronoyl moiety. **f**, **g** Two different views on aldouronic acid ligand recognition mode focusing on interactions with the xylo-oligosaccharide backbone. The superposition of the dΔS270A complexes with U^m4^X, U^m4^XX^-OH^, and XU^m4^XX^-OH^, based on the protein C_α_ atoms, illustrates little variation in the position of the ligand. Residues within 5 Å from the xylosyl moieties are labeled and shown in black. **h** dΔS270A:U^m4^XX^-OH^, aldotetrauronic acid in complex with the inactive variant. **i** Δ*dWT:U^m4^XX^-OH^, aldotetrauronic acid in complex with catalytically active *Cu*GE. Continuous electron density between the catalytic nucleophile S270 and the ligand suggests the formation of a covalent reaction intermediate.

Crystals obtained from dWT turned out to contain the apo form of the catalytic domain, resulting from cleavage of the wild-type enzyme (Δ*dWT, Fig. 1b). This shows that the deglycosylated, unprotected linker region is susceptible to proteolytic cleavage. The structure of Δ*dWT was determined to 1.96 Å resolution. The structure of the inactive truncated variant, apo-dΔS270A was determined to 1.46 Å resolution. Through soaking experiments with ligands mimicking parts of the natural substrate (Fig. 1a) it was possible to obtain high-resolution diffraction data for Δ*dWT and dΔS270A with ligands bound (Supplementary Tables 1 and 2, Fig. 3a–c, and Fig. 4). All crystals of the catalytic domain of *Cu*GE belong to the tetragonal space group *P*4_1_2_1_2 with two molecules in the asymmetric unit (Supplementary Fig. 3). The structure of apo-dΔS270A was determined by molecular replacement using Cip2 (3PIC)^9^ as search model.

The overall fold of the catalytic domain of *Cu*GE features a large ten-stranded twisted β-sheet flanked by α-helical elements and a surface-exposed active site (Fig. 3a). The structure complies with the characteristics of a serine esterase belonging to the α/β-hydrolase superfamily^11,23,24^. The electron density reveals a very well-defined domain comprising residues 81–458 (Figs. 1b and  3a–c). In all *Cu*GE structures, a single N-acetyl glucosamine moiety is attached to N104 as a relic of the N-glycosylation. In addition, signs of O-glycosylation (α-1-linked mannose) are observed in some of the structures (Fig. 3a).

The two crystallographically independent *Cu*GE molecules differ in their crystal packing environments and accessibility to the active site (Supplementary Fig. 3). Nevertheless, molecule A and B superimpose well with an RMSD of 0.32 Å for 359 aligned C_α_ atoms (apo-dΔS270A). Structures of ligand-bound complexes showed that the ligands bind in the highly accessible and solvent exposed binding site of molecule B only. Overall, molecule B is less tightly packed and the higher *B*-values suggest that some surface-exposed loops are more flexible in this molecule (Fig. 3b, c). It is noteworthy that the binding of ligands hardly induce any structural changes of the residues in the active sites.

### The canonical α/β-hydrolase fold of two major CE15 groups

*Cu*GE is the first structurally characterized representative of a CE15 enzyme from basidiomycetes and displays an overall architecture similar to Cip2^9^ and *St*GE2^12^ from ascomycetes. The structural differences between *Cu*GE and the two ascomycete’s enzymes relate to minor variations in length of loops and differences in the structure of the N-terminal residues, regions distantly located from the active site.

On the contrary, a comparison of CE15 structures in general shows distinct differences between fungal and bacterial members of the CE15 family (Supplementary Fig. 4). While all CE15 structures belong to the α/β-hydrolase superfamily^11,23,24^ scrutiny of their sequences and structures, coupled to the generally conserved structural zones of the active site of α/β-hydrolases^25,26^, shows that CE15 enzymes can be divided into two overall subgroups, CE15-A and CE15-B, corresponding to the catalytic acid configuration in the α/β-hydrolase group A and B, respectively (Fig. 3d–f). In CE15-A, the acidic member of the catalytic triad is located after β8 and is typically an aspartic acid (Fig. 3e). CE15-A comprises mostly bacterial enzymes, but also the fungal subgroup previously referred to as PPR8^6^ or G1^27^. In CE15-B, the catalytic acid is located after β7 and is always a glutamic acid. CE15-B comprises mostly fungal enzymes, including *Cu*GE, CiP2, and *St*GE2, but also enzymes of bacterial origin. Variations in the identity and position of the catalytic acid among members of CE15 have been discussed previously^6,7,13–15,18,27,28^. However, with our direct reference to the α/β-hydrolase superfamily we have been able to obtain a clear division of CE15 enzymes into two subgroups with fundamentally different properties encoded in their structural arrangements.

Besides the division in subgroups A and B based on the catalytic acid location and type, CE15 enzymes do not retain the canonical oxyanion zone observed in most α/β-hydrolases. The usual oxyanion hole conformation stabilizes the tetrahedral reaction intermediate by two main chain nitrogen atoms and involves a conserved H-G-x-X_oxyII_ motif at the end of β3 (β4 in CE15)^26^. CE15 structures show variations in this region; bacterial CE15-A members typically have an extra insert before αA, referred to as “insert 2”^13–15^ (Fig. 3e), whereas the fungal members of both CE15-A and CE15-B have a deletion and completely lack the canonical helix αA and the insert 2 (Fig. 3d–f and Supplementary Figs. 4–6). These differences have dramatic consequences for the overall shape of the substrate-binding site. A fundamental and unifying feature of both CE15 subgroups is the highly conserved CE15-signature motif GxSRxGK located in the nucleophilic elbow between β6 and αC (Supplementary Fig. 4) harboring a conserved arginine that seemingly play a role in the creation of the oxyanion hole^12,14^.

### Active site architecture and ligand-induced oxyanion hole

Consistent with the proven role in interfacial biocatalysis of derivatives of large LCC substrates^5,18^, the active site of *Cu*GE is readily accessible at the surface of the protein. The crystal structures (resolution 1.39–1.73 Å) of *Cu*GE complexes with aldouronic acids representing the carbohydrate portion of the natural substrate (XU^m4^X, Fig. 1a) significantly advance our knowledge on substrate recognition (Fig. 4a–i and Supplementary Tables 1 and 2). The active site appears remarkably robust with little variation between structures determined at different conditions (pH, active vs. inactive, apo vs. ligand-bound). Robustness of the active site region is a characteristic feature of α/β hydrolases^25,26^. In the fungal CE15 enzymes, this is reinforced by a conserved disulfide linkage that anchors two of the catalytic residues (H404 and S270 in *Cu*GE; Fig. 4b, c). Another characteristic feature is the very short hydrogen bond (2.5 Å) between E320 and E324 (Fig. 4e) that contributes to the creation of a relatively rigid active site, in which conformational variations are only observed for two residues: Y366 and R271. In apo-dΔS270A Y366 adopts a unique conformation in molecule B (pH 7.5–8; Fig. 4c) that seems to be incompatible with substrate binding, but identifies Y366 as a structurally flexible element. We observe that binding of aldouronic acids is coupled to a conformational change of R271 (Fig. 4c, d) that allows the side chain to engage in substrate recognition and complete the formation of the oxyanion hole. Thus, the oxyanion hole is created solely by R271 with both the backbone amide and guanidinium side chain nitrogen atoms forming close contacts with the O6 carbonyl of the 4-*O*-methyl-glucuronoyl moiety (3.2 and 2.8 Å, respectively), mimicking the interactions in a tetrahedral reaction intermediate (Fig. 4d–e). The unliganded structures of bacterial CE15 enzymes all display a pre-organized oxyanion hole with the arginine in an orientation very similar to the conformation observed in the ligand-bound structures of *Cu*GE. However, a corresponding conformational change of the conserved arginine was not observed in the *St*GE2 complex with the substrate analog methyl-4-*O*-methyl-glucuronic acid^12^.

### Recognition of the XU^m4^X-fragment

The aldouronic acid ligands dock onto the surface of *Cu*GE in all the complexes: dΔS270A:U^m4^X, dΔS270A:U^m4^XX^-OH^ and dΔS270A:XU^m4^XX^-OH^ without any major conformational changes of the slightly twisted, branched oligosaccharide. The α-1,2-linked 4-*O*-methyl-d-glucuronoyl moiety is nested in the small substrate-binding pocket, that provides distinct recognition of all hydroxyl groups and a hydrophobic environment for accommodation of the 4-*O*-methyl-d-glucuronoyl substituent, while the xylan-backbone runs along the surface and displays only hydrophobic, van der Waals and water-mediated interactions with the protein (Fig. 4d–g). The notion that no direct hydrogen bonds are involved in the recognition of the xylan-backbone is consistent with the variable structure of a natural LCC substrate with potential decorations at the C2- and C3-hydroxyl positions (Fig. 1a). Spatially, there is room for such decorations, except at the O3-position of the 4-*O*-methyl-glucuronoyl substituted xylosyl, where decoration could be unfavorable due to steric effects (clash with H404). In the complex formed at pH 7.5–8 (dΔS270A:U^4m^X), Y366 moves “out” upon substrate binding (Fig. 4c, d), and no significant differences are observed in substrate recognition interactions compared to the complexes formed at pH 5.5 (dΔS270A:U^m4^XX^-OH^ and dΔS270A:XU^m4^XX^-OH^) (Fig. 4f, g). All hydroxyl groups in the 4-*O*-methyl-d-glucuronoyl moiety are engaged in hydrogen bonds and the free carboxylate group displays polar interactions with both the catalytic H404 and R271 (Fig. 4d, e). R271 furthermore interacts with the O5 atom in the carbohydrate ring (Fig. 4d, e). Despite these extensive interactions with the 4-*O*-methyl-d-glucuronoyl moiety, soaks with the methyl ester of 4-*O*-methyl-glucuronic acid in concentrations up to 0.5 M did not result in a complex, indicating a low binding affinity for the ester and a clear preference for the larger aldouronic acid ligands. Indeed, the structure of U^m4^XX^-OH^ in complex with the active variant Δ*dWT shows continuous electron density, suggesting that a covalent acyl-enzyme intermediate has formed (Fig. 4i).

### *Cu*GE affinity for natural substrate and soluble fragments

**Fig. 5:**
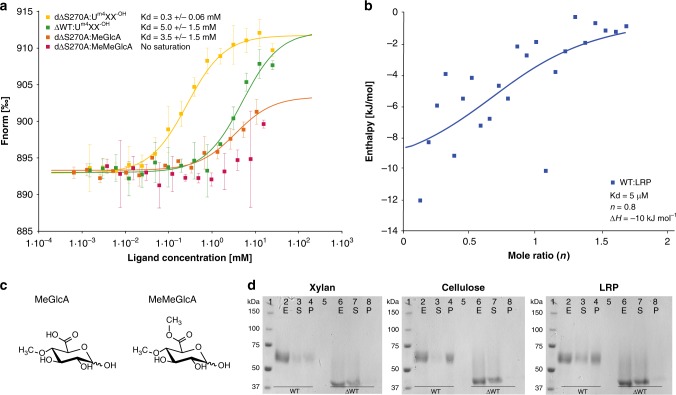
Affinity for natural substrates or derived soluble fragments. **a** Ligand-binding affinities of soluble ligands to various *Cu*GE variants as determined by LabelFree microscale thermophoresis (MST). Legend details and estimated *K*_d_-values are listed in the panel and error bars represents standard deviation. **b** ITC enthalpogram of the interaction between WT *Cu*GE and the enzyme’s natural insoluble lignin-rich substrate (LRP) at 5 °C. The titration curve is depicted as a function of the molar ratio (*n*) between enzyme and the calculated concentration of 4-*O*-methyl-glucuronoyl in the assay. The latter is an estimation of the concentration of recognition sites in the substrate. **c** Chemical structures of small ligands 4-*O*-methyl-d-glucuronic acid (MeGlcA) and the methyl ester of 4-*O*-methyl-d-glucuronic acid (MeMeGlcA) used in MST measurements of binding affinity in **a**. The structures are depicted to illustrate that equilibrium between the α- and β-anomers exists in an aqueous solution. **d** Qualitative SDS-PAGE-based “pull-down” assay illustrating the effect of CBM1 on the binding to the insoluble biopolymers xylan, cellulose and LRP, respectively. 1: Molecular size ladder, 2: WT without substrate, 3: Supernatant after binding between WT *Cu*GE and insoluble substrate, 4: Pellet after binding between WT *Cu*GE and insoluble substrate, 5: Insoluble substrate without enzyme, 6: ΔWT *Cu*GE without substrate, 7: Supernatant after binding between ΔWT *Cu*GE and insoluble substrate, 8: Pellet after binding between ΔWT *Cu*GE and insoluble substrate. The description of lane content applies to all three gel pictures. Source data for panel **a**, **b**, and **d** are provided as a Source Data file.

Binding affinity measured by microscale thermophoresis (MST) and isothermal titration calorimetry (ITC; Fig. 5a, b) shows differences in binding between soluble and insoluble ligands with a high affinity towards the insoluble substrate compared to soluble ligands and substrate analogs. MST measurements of ΔWT and dΔS270A respectively on aldotetrauronic acid (U^m4^XX^-OH^) indicate equilibrium dissociation constants in the mM range with a moderately stronger binding (one order of magnitude) between dΔS270A and U^m4^XX^-OH^ compared to the active variant. The strong binding in dΔS270A:U^m4^XX^-OH^ is in accordance with the well-defined binding observed in the complexes of *Cu*GE. The binding observed between ΔWT and U^m4^XX^-OH^ (*K*_d_ 5 mM) can be interpreted as a measure of the binding affinity of the enzyme to the carbohydrate moiety of the reaction product. Binding between dΔS270A and the 4-*O*-methyl-glucuronoyl moiety alone (structure shown in Fig. 5c) has ~10 times higher dissociation constant compared to dΔS270A:U^m4^XX^-OH^ and no saturation was obtained with the ester-mimicking model substrate methyl-4-*O*-methyl-d-glucuronic acid (structure shown in Fig. 5c), indicating a very low affinity (*K*_d_ > 10 mM). Besides the obvious lack of xylo-oligosaccharide backbone in the latter two ligands, neither of these two possesses a fixed α-anomeric configuration on C1, which may presumably bind stronger compared to the β-anomer. In nature, only the α-anomer have been found in glucuronoxylan and hence the binding may be optimized for this configuration. ITC measurements and model fitting of data points for binding between WT *Cu*GE and the insoluble lignin-rich precipitate from birchwood (LRP) rationalizes binding close to a 1:1 ratio with a relatively low enthalpy (~10 kJ mol^−1^) and a dissociation constant in the low µM range (Fig. 5b). The ITC titration data are corrected for background measurements from experiments without enzyme, and these controls reveal relatively high background signals, possibly related to use of an insoluble, particulate substrate. These background signals result in a low signal-to-noise ratio in the actual binding experiment, but the estimated *K*_d_ value (5 µM) imply a strong interaction between the enzyme and this insoluble substrate in comparison to those observed for soluble ligands. Previous kinetic studies with WT *Cu*GE on the same substrate^18^ revealed a *K*_M_ of ~220 µM and if *K*_M_ is taken as a measure of substrate affinity, the higher value compared to *K*_d_ measured by ITC suggests non-productive binding for instance by adsorption mediated by the CBM1 domain. Binding between *Cu*GE WT or ΔWT and the soluble ligands is too weak to be measured by ITC. Pull-down assays where WT and ΔWT *Cu*GE were incubated with insoluble xylan, cellulose or LRP (Fig. 5d) show that the full-length enzyme binds well to all three insoluble biomasses, whereas the truncated enzyme without its CBM1 binds less. In addition, *K*_M_ for ΔWT *Cu*GE on LRP is approx. twice as high compared to the full-length enzyme and *k*_cat_ is reduced by >30%^18^. Non-productive binding to lignin may occur in the presence of the CBM1 and could slow down the reaction speed. However, substrate recognition, binding and possibly also positioning of the enzyme to close-proximity cellulose or xylan is likely to be an important feature of *Cu*GE. Its location on the substrate may be an important factor for the main rate limiting step in natural, enzymatic conversion of LCCs.

### Conservation of carbohydrate recognition among fungal CE15s

The molecular details of XU^m4^X recognition and overall substrate-binding properties are likely to be generic for a large group of highly conserved fungal enzymes (Supplementary Fig. 5) where the sequence identity between CE15-B members are in the range of 41–77%. The fungal members of CE15-A display low overall sequence identity compared to the members of CE15-B, furthermore they differ in the configuration of the catalytic triad (Supplementary Figs. 5 and 6). The overall sequence identity within the fungal CE15-A group is 40–75%, whereas the sequence identity between the two groups is merely 25–36%.

**Fig. 6:**
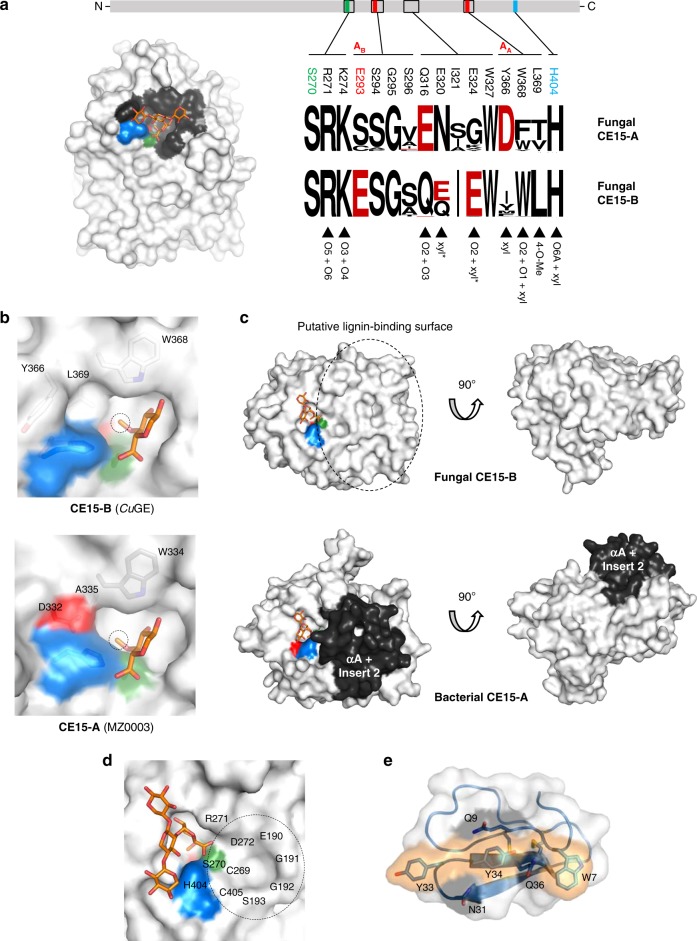
Conservation of structural features implicated in recognition of natural substrates by fungal CE15 enzymes. **a** Conservation of the carbohydrate-binding site. Residues within 5 Å from the XU^m4^X fragment are mapped onto the surface of *Cu*GE. Corresponding sequence logos^50^ are shown for the fungal subgroups CE15-A and CE15-B, respectively. The role(s) played by the individual residues in the *Cu*GE complexes with aldouronic acids are summarized. **b** Distinct environments around the 4-*O*-Methyl group caused by the different configurations of the catalytic triads in CE15-A and CE15-B. The ligand from the dΔS270A:XU^m4^XX^-OH^ structure is superimposed on the MZ0003 structure (6EHN)^13^. For clarity, only the 4-*O*-methyl-glucuronoyl-part of the ligand is shown. **c** Surface representations of *Cu*GE (CE15-B) and MZ0003 (CE15-A), illustrating differences in surface topology. Notably, the αA-helix and “insert 2” are only present in the bacterial members of CE15-A. In all fungal enzymes (both CE15-A and CE15-B), a deletion in this region results in a very open, flat, and accessible-binding site. **d** Putative monolignol binding site. **e** *Cu*GE-CBM1 homology model showing the conserved residues implicated in the adsorption onto insoluble substrates.

The variations between the substrate-binding sites in the two subgroups could reflect functional differences. To investigate this, a subset of residues that defines the XU^m4^X-binding site was selected and compared to the corresponding sequence logos produced from a large dataset of fungal CE15 sequences (Fig. 6a). Indeed, residues involved in carbohydrate recognition are almost invariant among fungal CE15-B enzymes and most of these residues appear to be conserved in fungal CE15-A members. Currently, there are no known structures of fungal CE15-A members. However, one notable difference (on sequence level) between CE15-A and CE15-B in their residues involved in substrate recognition is the lack of E324 in fungal CE15-A members (Supplementary Fig. 6). E324 interacts with O2, and could play a role in the specificity for the α-1,2-linked 4-*O*-methyl-glucuronoyl moiety. Noteworthy differences are also found at the position corresponding to Y366. In CE15-B, the latter is always a hydrophobic residue whereas CE15-A enzymes have their catalytic acid at this position. This creates distinct chemical differences between the two groups with respect to the properties of the pocket harboring the 4-*O*-methyl group (Fig. 6b) and could explain why CE15-A enzymes^14^, in contrast to CE15-B enzymes^16,17^, do not show a preference for 4-*O*-methylated substrates. A fungal CE15-A enzyme (*Tt*GE from *Thielavia terrestris*) has been shown to exhibit lower activity on LCC-containing substrate compared CE15-B enzymes, but prolonged incubation results in the same product profile as CE15-B enzymes^18^.

### Surface properties and implications for lignin interactions

Given the variable structure of lignin, a specific site for recognition of the alcohol moiety of the LCC substrate may not be present. Probably, the surfaces of GEs have evolved to have properties favorable for lignin interactions, rather than having conserved residues at specific positions. From a topological perspective we note that the *Cu*GE fold lacks the α/β hydrolase αA-helix (Fig. 3d–f), which leaves the fungal CE15 enzymes (both CE15-A and CE15-B) with a very open flat and accessible-binding site (Fig. 6c) that could allow interactions with ester substrates that have large and bulky alcohol fragments such as lignin. In *Cu*GE, this putative lignin binding surface is particularly rich in serine and threonine residues. In fungal CE15-B structures, a small depletion exists at the lignin-side of the ester cleavage site, which is conserved at the structural level (Fig. 6d). Conserved residues that might interact with a monolignol moiety are S193, C269, H404, and C405.

In the CBM1 domain, a number of highly conserved residues form a flat, aromatic binding site (Fig. 6e and Supplementary Fig. 2b), which is assumed to be implicated in the absorption onto insoluble substrates, including LRP. While the SAXS study of *Cu*GE shows an extended and relatively rigid linker region, the length of the linker varies significantly between different CE15 enzymes (Supplementary Fig. 7), indicating that a specific distance between the CBM1 and the catalytic domain is not crucial for the enzymatic function.

## Discussion

GEs have received much attention during the past 10 years, primarily due to their potential use in lignocellulose bioprocessing. The basis for understanding the action of GEs in heterogeneous biocatalysis relies on structural and functional knowledge of the enzymatic function. The fact that *Cu*GE has high affinity for the insoluble natural LCC-containing substrate LRP, compared to soluble ligands, implies that large lignin-rich substrates are the natural substrate structures for *Cu*GE. Our structural and functional studies of *Cu*GE have provided the structural basis for understanding the enzyme-substrate interactions and the well-defined complexes reveal how *Cu*GE recognizes and binds the carbohydrate moiety of the LCC. An interesting finding is that the 4-*O*-methylation occurring in natural glucuronoxylan is important for fitting the substrate into the active site of the enzyme. *Cu*GE possesses a flat open binding surface, where it is possible to accommodate even highly decorated xylans. In light of this binding mode, where the enzyme recognizes and binds the carbohydrate moiety of the LCC, it seems justified to conclude that the glucuronoyl esterases rightfully belong among the carbohydrate active enzymes in the CAZy database, acting on substrate on the interface between plant cell wall carbohydrates and the heterogeneous polyphenolic structures of lignin. The overall division of CE15s into two structurally different subgroups of α/β-hydrolases provides a clear and previously unnoted distinction between the CE15 enzymes and could indicate that the two subgroups evolved for two different enzymatic roles. While the fungal enzymes belonging to CE15-B appear highly conserved and specialized in recognizing and binding ester-linked LCCs from glucuronoxylan, the CE15-A variants seem to represent a subgroup of enzymes with broader substrate specificity and possibly even other types of activities, indicating that CE15-A enzymes have evolved to more generic types of esterases.

## Methods

### Preparation of lignin-rich precipitate from birchwood

LCC-containing substrate, originating from milled birchwood, was prepared by a mild organosolv type of extraction with 50 vol-% ethanol and 10% dry matter at 180 °C for 1 h. The liquid phase after organosolv extraction was precipitated with water to obtain a lignin-rich precipitate (LRP). A detailed method description is found in Mosbech et al.^5^. The LRP contains mainly lignin and minor amounts of covalently linked glucuronoxylan as seen from the compositional analysis of LRP, which showed that the precipitate contained ~90% Klason lignin and ~1.5% glucuronoxylan^5^. The concentration of 4-*O*-methyl-glucuronoyl residues was determined to be 2.39 mg g^−1^ dry matter, which corresponds to ~1·10^−2^ µmol 4-*O*-methyl-glucuronoyl mg LRP^−1^.

### Protein expression and purification

Three different variants of the CE15 glucuronoyl esterase *Cu*GE from *Cerrena unicolor* (Genbank accession number: MK422512) were recombinantly expressed in *Pichia pastoris* X-33; all with a C-terminal hexa histidine (His_6_) tag for affinity purification. (i) WT: full-length wild type, (ii) ∆WT: truncated *Cu*GE (residues G79-T458) corresponding to the catalytic domain only, and (iii) ∆S270A: inactive truncated variant where the catalytic nucleophile S270 was replaced by alanine. The ∆WT and ∆S270A constructs were designed with a TEV protease cleavage site upstream of the His_6-_tag to enable removal of the affinity tag from the purified protein. The genes encoding the *Cu*GE variants were codon-optimized for *P. pastoris*, prepared by GenScript and delivered in pPICZαA vectors. Different restriction sites were used for insertion of the constructs into the vector (*Eco*RI and *Xba*l for WT vs. *Eco*RI and *Sal*I for ∆WT and ∆S270A), resulting in a C-terminal extension with a *c-myc* epitope upstream of the His_6_ in the WT variant only. The three *Cu*GE variants were produced in *P. pastoris* and purified following the procedure described ealier^5^. The proteins were purified by affinity chromatography on an IMAC-column (HisTrap HP 5 mL column, GE Healthcare) using an Äkta Purifier 100 system (GE Healthcare, Uppsala Sweden).

### Deglycosylation

The affinity-purified samples displayed very smeared bands on sodium dodecyl sulfate–polyacrylamide gel electrophoresis (SDS-PAGE) indicating heterogenous N- and/or O-glycosylation^17^. Therefore, enzyme preparations to be used for structural studies were enzymatically deglycosylated and further purified by size-exclusion chromatography (SEC). In this procedure, the elution fractions from the IMAC step were immediately buffer exchanged into 20 mM sodium acetate pH 5 using PD-10 desalting columns (GE Healthcare) followed by concentration on Vivaspin 20 filter units with a 10 kDa MWCO (Sartorius). To trim the N-glycosylation at the single consensus site N104^17^, 42 mg purified ∆S270A were treated with 1U Endoglycosidase H (Endo H, Roche) at room temperature for 17 h in a total reaction volume of 8.5 mL. Similarly, 35 mg purified full-length WT enzyme was treated with 1U Endo H at room temperature in a total reaction volume of 9.8 mL. After 17 h of Endo H reaction, 260U α-1-2,3,6 mannosidase (P0768, New England Biolabs Inc.) was added in order to trim the O-glycosylation in the linker region^17^ and the deglycosylation reaction was allowed to proceed for a total of 52 h. The final purification of the deglycosylated samples, d∆S270A and dWT, was performed on a HiLoad 26/600 Superdex 75 column (GE Healthcare) equilibrated with 20 mM HEPES pH 7. Selected SEC fractions were pooled, concentrated to ∼10–15 mg mL^−1^ and stored at 4 °C until use. Protein concentrations were determined based on A_280_ measured with a Nanodrop 2000 (ThermoFisher Scientific) instrument and the calculated molar extinction coefficients (d∆S270A *ε*_280_ = 82765 M^−1^ cm^−1^; dWT *ε*_280_ = 101005 M^−1^ cm^−1^).

### Stability measurements

In order to evaluate the stability of the different *Cu*GE variants apparent unfolding temperatures (*T*_i_) were monitored based on changes in intrinsic fluorescence using a Tycho NT.6 instrument (NanoTemper Technologies). The measurements were made in 20 mM sodium acetate buffer pH 5, protein concentrations in the range 0.07–0.3 mg mL^−1^.

### Crystallization, data collection, and structure determination

The deglycosylated *Cu*GE variants dWT and dΔS207A were both screened for crystallization. However, all crystals obtained contain the catalytic domain only, as crystals formed in experiments with the full-length enzyme (dWT) turned out to be a proteolytic fragment lacking the CBM1 and linker region (Δ*dWT). Ligand complexes were prepared by soaking aldouronic acid ligands into crystals of either the inactive variant dΔS207A or the active variant Δ*dWT. Crystallization and ligand soak conditions are summarized in Supplementary Table 1. The vast majority of crystals formed at 4 °C and with high molecular weight polymers (SOKALAN CP7, PEG 10,000 or PEG 8000) as precipitants. Crystals were cryoprotected by brief soaks in either reservoir or ligand stock solutions supplemented with 26% glycerol and flash-frozen in liquid nitrogen. X-ray diffraction data were collected at cryogenic temperature (100 K) using the following beamlines: MASSIF-3 (ID30A-3) at ESRF, Grenoble, France (apo-dΔS207A, *λ* = 0.9677 Å); BioMAX at MAX IV, Lund, Sweden (apo-Δ*dWT, *λ* = 0.91837 Å); P13 at EMBL, DESY, Hamburg, Germany (dΔS207A:U^m4^X, *λ* = 0.97625 Å), ID29 at ESRF, Grenoble, France (dΔS207A:U^m4^XX^-OH^ and dΔS207A:XU^m4^XX^-OH^, *λ* = 0.99987 Å) and ID30B at ESRF, Grenoble, France (Δ*dWT:U^m4^XX^-OH^, *λ* = 0.97625 Å). Data collection and refinement statistics are given in Supplementary Table 2. The data sets were processed using XDS^29^. All crystals belong to the tetragonal space group P4_1_2_1_2 with two molecules in the asymmetric unit. The structure of apo-dΔS207A was solved by molecular replacement with PHASER^30^ using a trimmed version of the structure of Cip2 from *H. jecorina* (3PIC^9^, 54% seq. id.) as search model. The initial model was rebuilt and extended using the AutoBuild^31^ procedure in PHENIX^32^. The model was further rebuilt, extended and refined in iterative cycles with Coot^33^ and PHENIX^32,34^, respectively. A refinement protocol using the maximum-likelihood target, real-space refinement, refinement of coordinates, individual isotropic temperature factors and translation-liberation-screw (TLS) refinement using TLS groups determined by the PHENIX software was applied. All other *Cu*GE structures were solved by molecular replacement with PHASER using apo-dΔS207A as search model and refined in a similar manner as the apo-dΔS207A structure. All six *Cu*GE structures show the complete catalytic domain (C81-T458), whereas the terminal extensions are generally not ordered. However, the two structures obtained from the SOKALAN CP7 crystallization condition (apo-dΔS207A and dΔS207A:U^m4^X; Supplementary Table 1) are particularly well-defined and an N-terminal Glu-rich extension “EAEAEF” could be deduced from the electron density map. This sequence is derived from the pPICZαA expression vector and shows that apparently only *kex*2 cleavage, but not STE13 cleavage, has occurred when recombinant *Cu*GE was produced in the *P. pastoris* expression system. All structures contain one NAG residue at N104, while the two structures originating from crystallization experiments with full-length *Cu*GE (apo-Δ*dWT and Δ*dWT:U^m4^XX^-OH^) additionally contain a MAN residue at both S86 and T87, indicating that these sites only get O-glycosylated when the full-length enzyme is expressed. Soaks with aldouronic acids resulted in very well-defined complexes with the ligand-bound in the more accessible molecule B only. In the dΔS207A:U^m4^X structure, which was obtained by soaking with a mixture of aldouronic acids (O-AMX, Megazymes), positive difference density suggested the presence of a partially occupied xylose moiety at the reducing end of the U^m4^X ligand. This residue was, however, not included in the final model as the occupancy was too low to achieve a robust refinement. The Δ*dWT:U^m4^XX^-OH^ structure displayed continuous electron density between the catalytic nucleophile S270 and C6 of the aldouronic acid ligand and was therefore modeled as a covalent reaction intermediate; the S270(OG)-C6 distance refined to 1.62 Å. An ethylene glycol (EDO) molecule was observed in the substrate-binding pocket of all unliganded structures (molecule A and apo-structures). At the final stages of refinement, positive difference density suggested the presence of a secondary surface binding site in the structures of dΔS207A:U^m4^XX^-OH^ and Δ*dWT:U^m4^XX^-OH^. This was modeled as a partially occupied xylobiose molecule (BXP). The data to high resolution and the complex composition of the solvent region comprising water, glycerol, ethylene glycol molecules, and the protein side chains in weakly populated alternative conformations made it non-trivial to model of the electron density in the solvent region. Only fully occupied solvent molecules were included in the final models, however, a number of partial occupied solvent molecules are visible in the electron density maps.

The Ramachandran statistics calculated by MolProbity^35^ for the final refined models are as follows: 96.3% in the favored region, 3.7% allowed, 0% outliers for apo-dΔS207A; 96.0% in the favored region, 4.0% allowed, 0% outliers for apo-Δ*dWT; 96.2% in the favored region, 3.8% allowed, 0% outliers for dΔS207A:U^m4^X; 96.1% in the favored region, 3.9% allowed, 0% outliers for dΔS207A:U^m4^XX^-OH^; 96.3% in the favored region, 3.7% allowed, 0% outliers for dΔS207A:XU^m4^XX^-OH^ and 96.3% in the favored region, 3.7% allowed, 0% outliers for Δ*dWT:U^m4^XX^-OH^, respectively. Illustrations were prepared with PyMOL (http://www.pymol.org).

### Small-angle X-ray scattering

Prior to the analysis by small-angle X-ray scattering (SAXS), the deglycosylated variants of full-length (dWT) and truncated, inactive *Cu*GE (dΔS207A) were diafiltered into 20 mM sodium acetate pH 5 and centrifuged at 14,000 × *g* for 10 min at 4 °C. The permeates were used as reference solutions. For each protein, three different dilutions were prepared in 20 mM sodium acetate buffer pH 5 (dWT 3.7, 2.0, and 1.0 mg mL^−1^ and dΔS207A 3.6, 2.0 and 1.0 mg mL^−1^). SAXS measurements were performed on a Xenocs BioXolver L equipped with a GeniX3D microfocus X-ray source (*λ* = 1.54 Å) and a BioCUBE sample environment. A sample-to-detector distance of 571 mm was used, corresponding to values of the scattering vector *q* between 0.019 and 0.48 Å^−1^. Samples were kept and measured at room temperature, automatic loading was done using the sample handling robot from a 96-well tray. Scattering data was collected as 10–20 frames (depending on sample concentration) of 60 s exposure pr. frame for protein samples, as well as corresponding buffers. Two-dimensional (2D) images were radially averaged, and overlap of individual frames ensured before merging. Buffer scattering was subtracted from the total scattering. In addition, a bovine serum albumin (BSA) solution was measured for reference and molecular weight estimation. All initial data processing was done using the software RAW^36,37^. Subsequent primary data analysis, Guinier fit, indirect Fourier transformation and molecular weight estimation, was done using Primus^38^. For dΔS207A the solution scattering data was compared to the crystal structure of apo-dΔS207A (molecule A) using CRYSOL^39^. Ab-initio shape reconstruction based on the data from dWT was performed using both DAMMIF^40^ and GASBOR^41^, in both cases using the *q* range from 0.02 to 0.25 Å^−1^. DAMMIF was run in default slow mode generating ten models. After alignment (mean NSD = 0.612), the models were averaged and refined in DAMMIN^42^. GASBOR was run with default settings fitting intensity in reciprocal space. Illustrations of the structural models were prepared with PyMOL (http://www.pymol.org).

### Homology model

A homology model of *Cu*GE-CBM1 (residues 3–38) was made with SWISS-MODEL^43^ using the structure of the CBM1 from *Trichoderma reesei* cellobiohydrolase I (PDB code 1CBH^44^) as template. The sequence identity between *Cu*GE-CBM1 and 1CBH is 64%.

### Microscale thermophoresis

Binding affinities for the soluble ligands U^m4^XX^-OH^ (O-UXXR, MEGAZYMES), MeGlcA (4-*O*-methyl-d-glucuronic acid, MM46686, Carbosynth, Crompton UK), and MeMeGlcA (4-*O*-methyl-d-glucopyranosyluronate, purchased from Institute of Chemistry, Slovak Academy of Sciences, Bratislava, Slovakia) were measured by MST. The protein stocks, dΔS207A and ΔWT, were adjusted to 200 nM with 100 mM sodium acetate buffer pH 5 supplemented with 0.1% Pluronic F-127. Ligand stocks (100 mM U^m4^XX^-OH^, 43 mM MeGlcA and 250 mM MeMeGlcA) were prepared in 100 mM sodium acetate buffer pH 5, and for each ligand a series of 16 1:1 dilutions was prepared using the same buffer. For the measurement, each ligand dilution was mixed with one volume of protein, which led to a final protein concentration of 100 nM and final ligand concentrations in the mM to nM range. After 10 min incubation followed by centrifugation at 10,000 × *g* for 10 min, the samples were loaded into Monolith NT.LabelFree zero-background capillaries (NanoTemper Technologies). The MST experiment was performed with a Monolith NT.LabelFree instrument (NanoTemper Technologies) at 25 °C using 30% LED power and medium MST power. For MeGlcA and MeMeGlcA, the higher ligand concentrations resulted in significant variations in raw fluorescence and these data were therefore not included in the analysis. Final ligand concentration ranges used in the analysis of the MST signal were: U^m4^XX^-OH^ 25 mM to 1.53 μM, MeGlcA 10.8 mM to 656 nM and MeMeGlcA 15.6 mM to 3.81 μM. For each ligand-binding curve, data from at least three independently pipetted measurements were included in the analysis with the MO. Affinity Analysis software (version 2.3, NanoTemper Technologies) using the signal from an MST-on time of 5 s.

### Isothermal titration calorimetry measurements

Binding affinity of full-length WT *Cu*GE to the insoluble lignin-rich precipitate (LRP) from birchwood was determined by ITC by measuring the heat generated upon binding of the enzyme to a ligand, in this case LRP. The measurements were performed on a NanoITC 2G instrument from TA instruments (Lindon, USA) equipped with a 950 µL titration cell and an equal size reference cell. The reference cell was loaded with 20 mM MES buffer pH 6 throughout all titrations. Prior to ITC experiments, the WT *Cu*GE preparation was buffer exchanged into 20 mM MES buffer pH 6 on a spin filter with a polyethersulfone membrane with a 10 kDa MWCO (Vivaspin 20, Sartorius). The buffer was exchanged three times prior to final round of ultrafiltration. Permeate from the last round was used as suspension buffer for LRP in the titration experiments to obtain near-identical environments in cell and syringe. Nine-hundred fifty microliters LRP-suspension (3.5 mg mL^−1^, corresponding to ~0.04 mM 4-*O*-methyl-glucuronoyl residues) in permeate was degassed for 5 min and loaded directly to the titration chamber. The sample syringe was loaded with 250 µL 0.2 mM WT preparation and the syringe needle was placed inside the titration cell. The needle of the sample syringe was equipped with impellers for constant rotational stirring during the titration and stirring speed was set to 300 rpm. The titration was performed at 5 °C, where hydrolysis was found to be negligible within the time frame of the assay and the enzyme preparation was added over the course of 25 injections of 10 µL each. The time intervals between each injection was 300 s and prior to the first injection the system was allowed to equilibrate until steady baseline was achieved, however no longer than 60 min. A control experiment with permeate buffer in the sample syringe instead of enzyme preparation was performed with the same settings to assess the level of heat generation not associated with enzyme-ligand binding. The results of the enzyme-ligand measurements were corrected for background heat generation by subtracting the control measurements prior to data handling. All data handling was performed using NanoAnalyze Data Analysis v. 3.8.0 provided by TA instruments and model fitting was performed by the use of an independent data model provided by the software.

### Binding to insoluble biopolymers, pull-down assay

To assess the effect of the CBM1 module on the binding of *Cu*GE to insoluble biopolymers a pull-down assay was performed. Suspensions of 10 mg mL^−1^ biopolymer, including Avicel® PH-101 cellulose (Sigma-Aldrich), xylan from birchwood (Sigma-Aldrich), and LRP, were prepared in 50 mM sodium acetate buffer pH 5. Full length and truncated *Cu*GE variants, WT and ∆WT, respectively, were added to a final concentration of 0.07 mg mL^−1^ in a total reaction volume of 550 µL. The samples were incubated for 1 h at 4 ^◦^C with rotation. After incubation the reaction tubes were centrifuged at 14,000 rpm for 10 min to separate pellet and supernatant. The pellet, containing protein bound to the insoluble biopolymer, was washed in water twice and then re-suspended in 550 µL water. To increase the protein concentration all samples were freeze dried and re-suspended in 100 µL prior to analysis. Samples were reduced with DTT and analyzed by SDS-PAGE using Mini-PROTEAN® TGX™ Precast Gels (Bio-RAD).

### Topology diagrams and CE15 subgroups

The topology diagrams were made in PowerPoint based on the description of the classical α/β-hydrolase fold^23–25^ combined with a manual inspection of all structurally characterized CE15 enzymes (PDB codes 6RTV, 4G4G^12^, 3PIC^9^, 6EHN^13^, 6GS0^14^, 6GRY^14^, and 6HSW^15^). The feruloyl esterase Est1 from *Butyrivibrio proteoclasticus* (PDB code 2WTM^45^) was included as a reference for an α/β-hydrolase Group A fold and a canonical oxyanion hole. With reference to the α/β-hydrolase Groups A and B^25^, the CE15 members were assigned to the subgroups CE15-A or CE15-B, respectively, depending on the position of the catalytic acid after either strand β8 (CE15-A) or β7 (CE15- B). The structure of the bacterial MZ0003^13^ was used as representative for CE15-A, as no fungal CE15-A member has so far been structurally characterized, and the *Cu*GE structure was used as representative of CE15-B. For clarity, only the major secondary structural elements were included in the topology diagrams. Structures from both CE15 subgroups contain additional small secondary structural elements, encompassing multiple helical elements inserted after the strands β5 and β7 (Supplementary Fig. 4). The bacterial CE15-A structures comprise the canonical α/β-hydrolase helix αA, as well as the three inserts I1, I2, and I3. However, a comparison with the sequence alignments in Supplementary Figs. 4–6 suggests that these structural elements are not present in the fungal CE15-A members.

### Sequence analysis

Multiple sequence alignments were made with ProMALS3D^46,47^, further edited by hand and visualized using Jalview^48^. The sequence logos, were based on a dataset corresponding to the sequences used by Dilokpimol^49^ complemented with the *Afu*GE sequence^18^. A subset of binding site residues was defined, within 5 Å from the XU^m4^X fragment in the dΔS207A:XU^m4^XX^-OH^ structure (PDB code 6RV9), and residues at the corresponding positions were extracted from the alignment comprising all fungal CE15 sequences in the dataset (Supplementary Figs. 5 and 6). Sequence logos were produced with WebLOGO^50^ using a total of 20 sequences for the fungal subgroup CE15-A and 116 sequences for the fungal subgroup CE15-B, respectively.

### Reporting summary

Further information on research design is available in the Nature Research Reporting Summary linked to this article.

## Supplementary information

Supplementary Information

Peer Review

Reporting Summary

## Data Availability

The data that support this work are available from the corresponding authors upon reasonable request. The SAXS data and corresponding models presented in the manuscript has been deposited at SASBDB (www.sasbdb.org) with accession codes: SASDGD6 and SASDGC6. The crystal structures presented in this manuscript have been deposited in the Protein Data Bank with the PDB codes 6RTV, 6RU1, 6RU2, 6RV7, 6RV8 and 6RV9, respectively. It must be noted that the SAXS and the PDB models differ in their sequence numbering. The SAXS model use UNIPROT numbering, whereas the PDB models refer to the sequence numbering illustrated in Fig. 1. The source data underlying Fig. 1c, 5a–d, and Supplementary Fig. 1 are provided as a Source Data file.

## Source data

Source Data
