# Supplementary material for: The structural basis of fungal glucuronoyl esterase activity on natural substrates
Source: Nat Commun. 2020 Feb 24;11:1026. doi: 10.1038/s41467-020-14833-9 (PMC7039992; doi:10.1038/s41467-020-14833-9)
Supplement: Supplementary file 1 — Supplementary Information [file 41467_2020_14833_MOESM1_ESM.pdf]

Supplementary Information

The structural basis of fungal glucuronoyl esterase activity on natural substrates

Ernst et al.

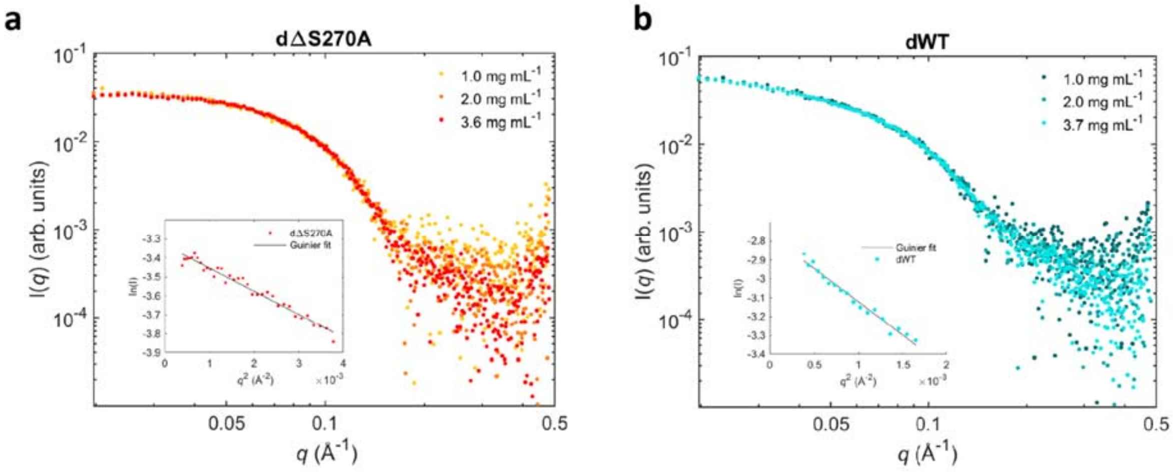

c

	$Rg_{Guinier}$ (Å)	$Rg_{P(r)}$ (Å)	D_{max} (Å)	MW_{BSA} (kDa)	MW_{BI} (kDa)	MW_{theo} (kDa)
dΔS270A	20.1 +/- 0.8	20.5	60	33	35 (32-35)	42.7
dWT	31.9 +/- 2.9	32.2	110	55	45 (43-47)	50.9

Supplementary Figure 1 – SAXS data

a) Experimental SAXS scattering profiles from dΔS270A measured at different concentrations. Insert: Guinier fit to the 3.6 mgmL^{-1} data. **b**) Experimental SAXS scattering profiles from dWT. **c**) Summary of parameters from the primary SAXS data analyses. Two different approaches are included for MW estimation. MW_{BSA} is the classical approach, being concentration dependent and based on measurements of Bovine Serum Albumin (BSA) as reference. MW_{BI} is a recent addition to PRIMUS, being concentration independent and based on Bayesian Inference[1]. Confidence intervals are given in parentheses. Source data for panel a and b are provided as a Source Data file.

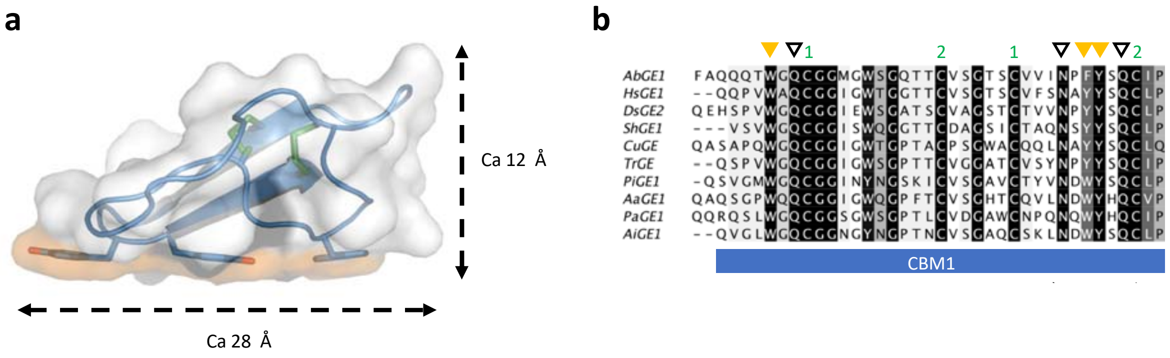

Supplementary Figure 2 – *Cu*GE-CBM1 homology model

a) Homology model of *Cu*GE-CBM1 (residues 3-38) made with SWISS-MODEL[2] using the structure of 1CBH[3] as template. The CBM1 domain is wedge-shaped with approximate dimensions 28 Å x 12 Å (thick end) x 20 Å. The fold is stabilized by two disulfide bridges (green) and three conserved aromatic residues (orange) create a flat binding site expected to be implicated in binding of insoluble substrates. **b**) Multiple sequence alignment of CBM1 domains from functionally characterized fungal CE15 enzymes. Conserved residues forming the flat binding site are marked by orange triangles for aromatic residues and open triangles for polar residues. Cysteines involved in disulphide bridges are marked by green numbers.

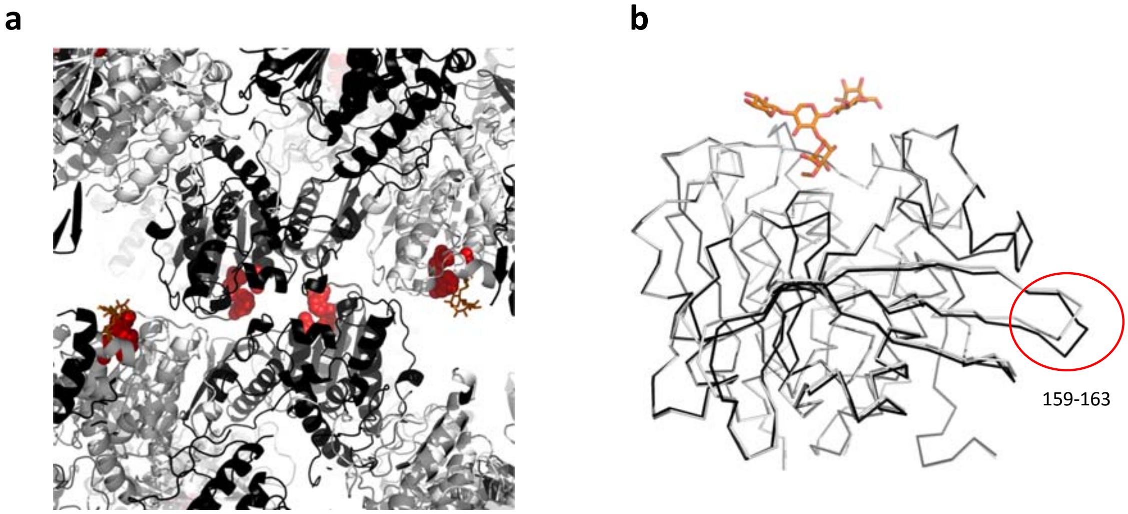

Supplementary Figure 3 – *Cu*GE crystal packing and two distinct active site environments

a) Illustration of the crystal packing in the tetragonal space group $P4_12_12$. The two crystallographically independent molecules A (black) and B (white) differ in crystal packing environments and accessibility to the active site. To illustrate the location of the active site, the catalytic residues are shown as red spheres and the ligand from the ΔS270A:XU^{m4}XX^{-OH} complex is shown in orange. Note that the active site in molecule A is partly occluded due to contacts in the crystal with a symmetry related molecule (A'), whereas the binding site in molecule B is solvent-exposed and easily accessible. **b**) Superposition of molecules A (black) and B (white) shown as C_α-traces. Molecule A and B superimpose with an RMSD of 0.32 Å for 359 aligned C_α atoms (apo-dDS270A), reflecting only minor structural variations. The main difference concerns the β-turnb2-b3 (residues 159-163), which is affected by crystal packing of molecule B.

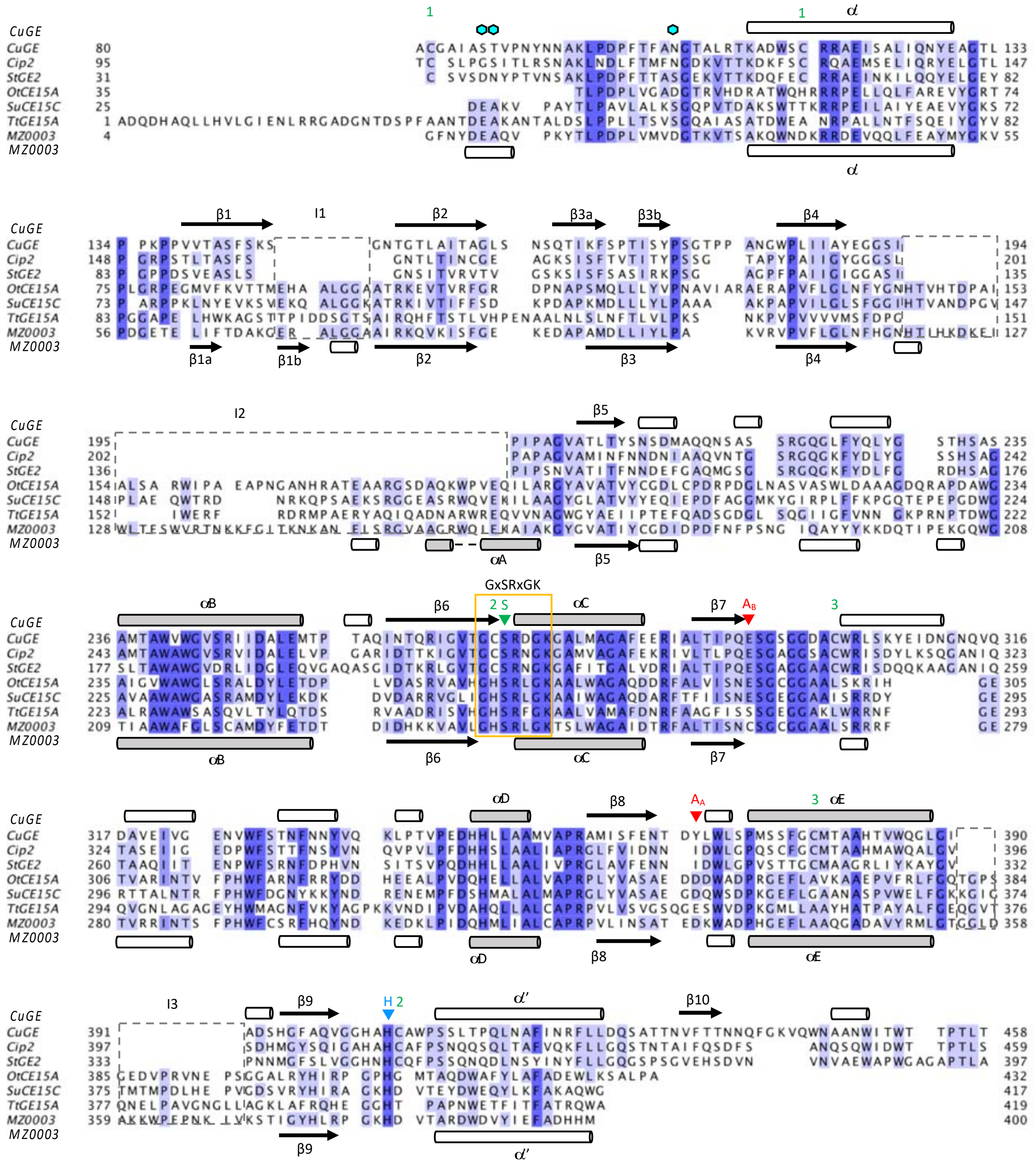

Supplementary Figure 4 – Structure-based sequence alignment of all structurally characterized CE15 enzymes. Secondary structure elements are shown for MZ0003 (6EHN)[4] and *Cu*GE (6RTV) as representatives for subgroups CE15-A and CE15-B, respectively. The major secondary structure elements are labelled according to the topology diagrams in Fig. 3 d-f in the main text. Residues comprising the catalytic triad are indicated with triangles: nucleophile (serine) in green, base (histidine) in blue and acid (glutamate/aspartate) in red. The two different structural locations of the acidic residue are labeled A_A or A_B, with reference to the configuration in α/β-hydrolase group A and B, respectively. The three major "inserts" in the bacterial structures are labelled I1, I2 & I3, green numbers indicated disulfide bridges and cyan hexagons symbolize glycosylation sites observed in the *Cu*GE structures. The CE15-signature sequence "GxSRxGK" is highlighted with an orange box. PDB codes for all structures in the alignment: *Cu*GE 6RTV; Cip2 3PIC; *St*GE2 4G4G; *Ot*CE15A 6GS0; *Su*CE15C 6GRY; *Tt*GE15A 6HSW, MZ0003 6EHN.

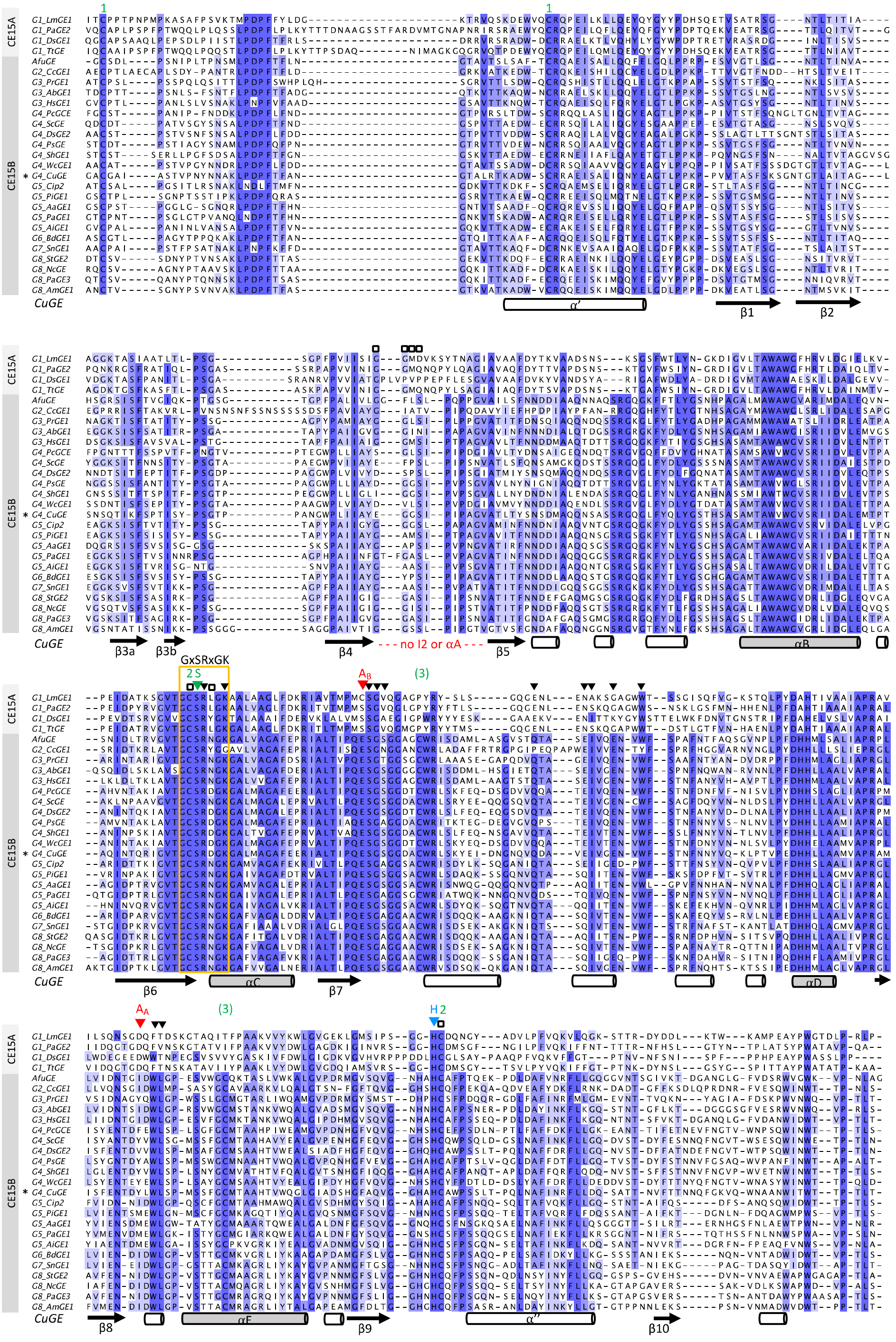

Supplementary Figure 5 Multiple sequence alignment (MSA) of functionally characterized fungal CE15-A and CE15-B enzymes. The alignment comprises sequences included in the nomenclature paper by Dilokpimol *et al*. on fungal CE15 GEs (subgroups G1-G8)[5] and the *Afu*GE sequence from Mosbech *et al*.[6] Catalytic residues are indicated with triangles as in Fig. 3 and Supplementary Fig. 4. Other residues within 5 Å from the XU^{m4}X moiety in the *Cu*GE:ligand complexes are marked with black triangles. Residues forming the putative monolignol docking site are marked with open squares. The two different structural locations of the acidic residue, that differentiate the subgroups CE15-A and CE15-B, are labeled A_A and A_B, respectively. Secondary structure elements from the *Cu*GE structure (6RTV) are included for reference. The MSA suggests that the fungal CE15-A enzymes, apart from the distinct location of the acidic catalytic residue, display considerable structural similarity to their CE15-B counterparts. Two of the three disulfide bridges (green numbers) are conserved. Moreover, the loop between β4 and β5 is only a few residues longer in fungal CE15-A enzymes implying that these enzymes also lack I2 and the αA-helix and thus are predicted to have a relatively open binding site similar to fungal CE15-B enzymes.

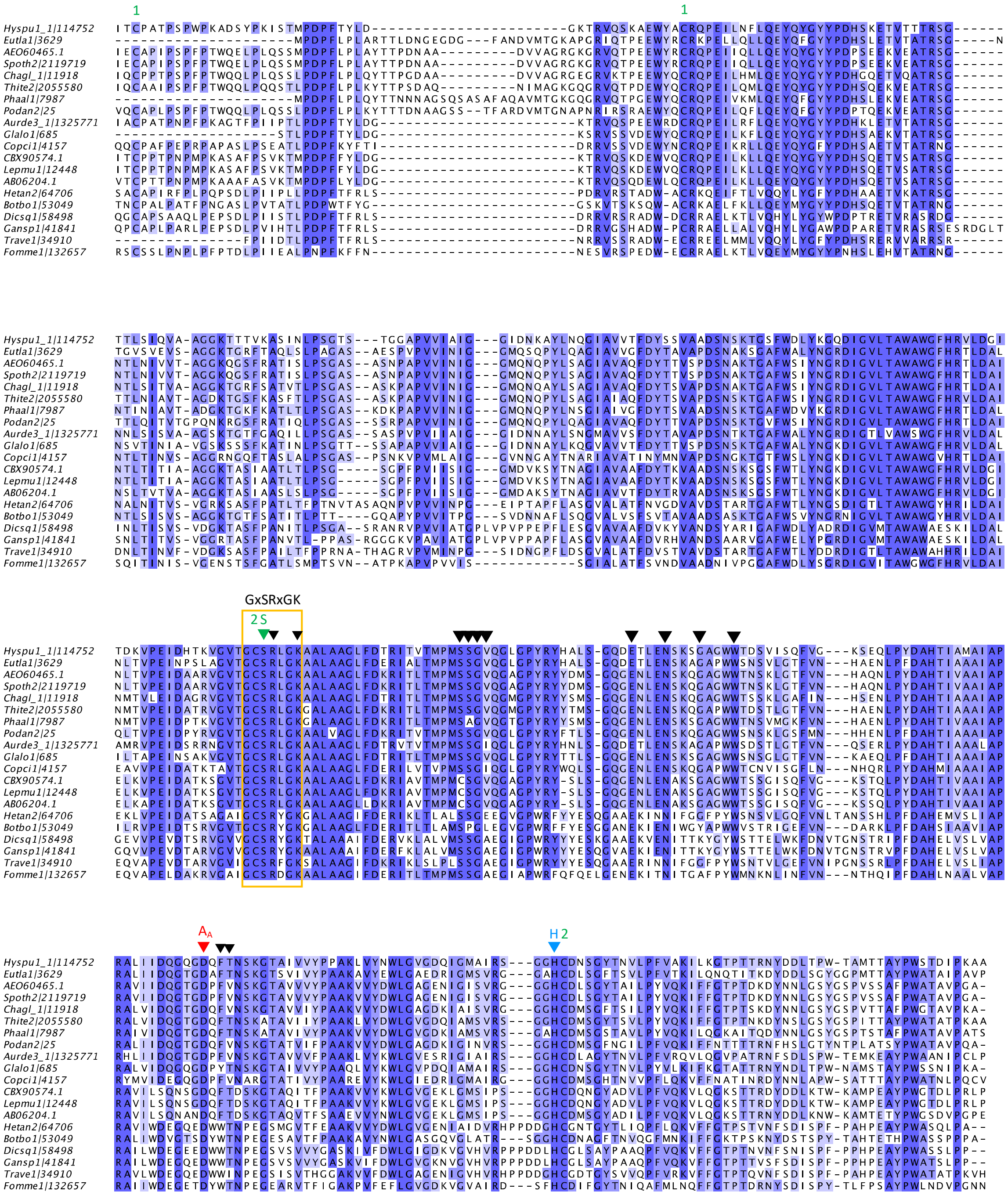

Supplementary Figure 6 Multiple sequence alignment of the fungal CE15-A members, including non-characterized sequences. To further probe the variability among fungal CE15-A enzymes, all sequences assigned to the G1 subgroup in Dilokpimol *et al.*[5] were included in the alignment. This small fungal subgroup appears highly conserved at sequence level, except for an insert present in the N-terminal region of some members only. Based on the structures of *Cu*GE:ligand complexes it is predicted that the substrate binding site is formed by the catalytic triad (colored triangles) and residues at the positions marked by black triangles. The natural substrate(s) of fungal CE15-enzymes remains speculative.

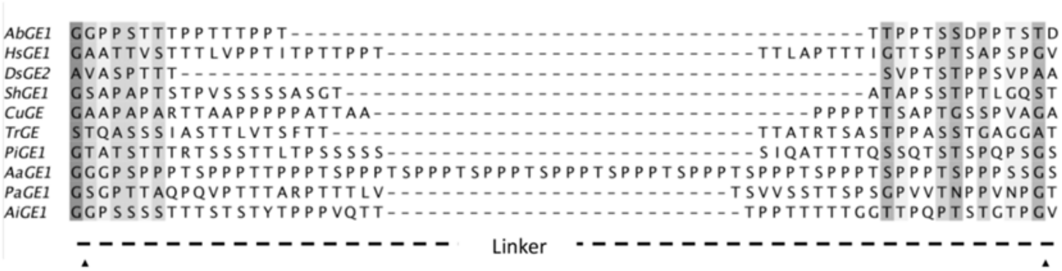

Supplementary Figure 7: Multiple sequence alignment of linker regions between catalytic and carbohydrate binding modules of fungal CE15 enzymes. Linker regions from functionally characterized two-domain CE15 enzymes display significant variation in length and amino acid composition. Putative hinge regions at either end of the linker are indicated.

Supplementary Table 1 – Crystallization and soak conditions for the *Cu*GE structures

	**apo-dΔS270A**	**apo-Δ*dWT**	**dΔS270A:$U^{m4}X$**	**dΔS270A:$U^{m4}XX^{-OH}$**	**dΔS270A:$XU^{m4}XX^{-OH}$**	**Δ*dWT:$U^{m4}XX^{-OH}$**
PDB accession code	6RTV	6RU2	6RU1	6RV7	6RV9	6RV8
Vapor diffusion setup	Hanging drop	Sitting drop	Hanging drop	Sitting drop	Sitting drop	Sitting drop
Protein concentration (mg mL^{-1})	10	10	5	10.9	10.9	10
Reservoir condition	0.1 M HEPES pH 7, 0.1 M KCl, 25 % w v^{-1} SOKALAN CP7	0.1 M ammonium acetate, 0.1 M bis-tris pH 5.5, 17 % w v^{-1} PEG10000	0.1 M HEPES pH 7, 0.1 M KCl, 25 % w v^{-1} SOKALAN CP7	0.1 M phosphate-citrate pH 4.2, 0.1 M KCl, 20 % w v^{-1} PEG8000	0.1 M phosphate-citrate pH 4.2, 0.2 M KCl, 20 % w v^{-1} PEG8000	0.1 M ammonium acetate, 0.1 M bis-tris pH 5.5, 17 % w v^{-1} PEG10000
Drop volume (μL)	2	0.4	3	0.4	0.4	0.4
Ratio (protein:reservoir)	1:1	3:1	2:1	3:1	3:1	3:1
Temperature (°C)	4	4	4	4	4	4
Ligand			$U^{m4}X$	$U^{m4}XX^{-OH}$	$XU^{m4}XX^{-OH}$	$U^{m4}XX^{-OH}$ covalent intermediate
Product ID			O-AMX	O-UXXR	O-XUXXR	O-UXXR
Manufacturer			Megazyme	Megazyme	Megazyme	Megazyme
Soak condition			Ligand stock (25 mg mL^{-1}) in reservoir solution was added 1:1 to the drop	Ligand stock (252 mM in 0.1 M Na_3-citrate pH 5.5, 0.2 M KCl, 20 % w v^{-1} PEG8000) was added 7:1 to the drop	Ligand stock (305 mM in 0.1 M Na_3-citrate pH 5.5, 0.2 M KCl, 20 % w v^{-1} PEG8000) was added 7:1 to the drop	Ligand stock (253 mM) in reservoir solution was added was added 1:1 to the drop
Soaking time (min)			60	45	50	60
Cryo condition	Reservoir condition supplemented with 26 % glycerol	Reservoir condition supplemented with 26 % glycerol	Ligand stock supplemented with 26 % glycerol	Ligand stock supplemented with 26 % glycerol	Ligand stock supplemented with 26 % glycerol	Ligand stock supplemented with 26 % glycerol

Supplementary Table 2 – Data collection and refinement statistics for the *Cu*GE structures

	apo-dΔS270A	apo-Δ*dWT	dΔS270A:U^{m4}X	dΔS270A:U^{m4}XX^{-OH}	dΔS270A:XU^{m4}XX^{-OH}	Δ*dWT:U^{m4}XX^{-OH}
PDB accession code	6RTV	6RU2	6RU1	6RV7	6RV9	6RV8
**Data collection**						
Space group	$P\ 4_12_12$	$P\ 4_12_12$	$P\ 4_12_12$	$P\ 4_12_12$	$P\ 4_12_12$	$P\ 4_12_12$
Cell dimensions						
a=*b*, *c* (Å)	84.27, 261.13	84.45, 262.24	84.24, 260.82	84.78, 260.38	84.48, 261.73	84.75, 261.06
Resolution (Å) [a]	44.0 - 1.46 (1.51 - 1.46)	44.2 - 1.96 (2.03 - 1.96)	42.1 - 1.39 (1.44 - 1.39)	49.3 - 1.73 (1.79 - 1.73)	44.5 - 1.64 (1.70 - 1.64)	44.1 - 1.85 (1.92 - 1.85)
R_{merge} [b]	0.158 (0.827)	0.252 (0.852)	0.103 (0.844)	0.167 (1.04)	0.081 (0.867)	0.106 (0.859)
$\langle I / \sigma I \rangle$	7.99 (1.90)	6.64 (1.72)	11.96 (1.78)	10.39 (1.56)	13.85 (1.68)	28.48 (3.78)
Wilson B-value (Å^2)	11.5	20.1	12.5	20.0	23.3	24.6
Unique reflections	163501 (16134)	68186 (6594)	188193 (18515)	99921 (9775)	116768 (11435)	80254 (6618)
Completeness (%)	100.0 (100.0)	98.2 (97.1)	99.5 (99.3)	99.8 (100.0)	99.5 (99.4)	97.6 (82.0)
Redundancy	9.7 (8.8)	8.7 (8.5)	7.4 (7.3)	7.0 (7.1)	8.1 (7.6)	24.9 (19.3)
$CC_{1/2}$	0.997 (0.555)	0.989 (0.665)	0.996 (0.693)	0.997 (0.675)	0.998 (0.683)	0.999 (0.856)
**Refinement**						
Resolution (Å)	44.0 - 1.46	44.2 - 1.96	42.1 - 1.39	49.3 - 1.73	44.5 - 1.64	44.1 - 1.85
No. reflections	163458 (16133)	67954 (6564)	188018 (18507)	99779 (9773)	116315 (11427)	80246 (6618)
R_{work} / R_{free}	0.182/0.199	0.178/0.204	0.163/0.177	0.166/0.194	0.186/0.209	0.150/0.181
No. atoms						
Protein	5936	5764	5954	5780	5738	5771
Ligand/ion	79	87	107	92	82	135
Water	757	542	734	518	527	579
B-factors (Å^2)						
Protein	13.7	20.1	14.7	22.9	31.0	25.6
Ligand/ion	24.6	39.0	23.7	30.5	31.9	47.1
Water	24.7	26.7	25.6	29.3	33.2	34.2
R.m.s. deviations [c]						
Bond lengths (Å)	0.01	0.01	0.01	0.03	0.03	0.03
Bond angles (°)	1.2	1.1	1.2	1.6	1.6	1.6
Ramachandran (%)						
Favored	96	96	96	96	96	96
Allowed	4	4	4	4	4	4
Outliers	0	0	0	0	0	0
Number of TLS groups	10	17	11	8	10	13

One crystal was used for each structure. *Values in parentheses are for the highest-resolution shell. [b] $R_{merge} = \sum_{hkl}\sum_{i=1}^{n}|I_i\ (hkl) - \bar{I}\ (hkl)|\ /\ \sum_{hkl}\sum_{i=1}^{n} I_i\ (hkl)$. [c] R.m.s. deviations from the ideal values reported by Engh & Huber[7]

REFERENCES

1. Hajizadeh, N. R., Franke, D., Jeffries, C. M. & Svergun, D. I. Consensus Bayesian assessment of protein molecular mass from solution X-ray scattering data. *Sci. Rep.* **8**, 7204 (2018).

2. Waterhouse, A. *et al.* SWISS-MODEL: homology modelling of protein structures and complexes. *Nucleic Acids Res* **46**, W296–W303 (2018).

3. Kraulis, J. *et al.* Determination of the three-dimensional solution structure of the C-terminal domain of cellobiohydrolase I from Trichoderma reesei. A study using nuclear magnetic resonance and hybrid distance geometry-dynamical simulated annealing. *Biochemistry* **28**, 7241–7257 (1989).

4. De Santi, C., Gani, O. A., Helland, R. & Williamson, A. Structural insight into a CE15 esterase from the marine bacterial metagenome. *Sci. Rep.* **7**, 17278 (2017).

5. Dilokpimol, A. *et al.* Fungal glucuronoyl esterases: Genome mining based enzyme discovery and biochemical characterization. *N. Biotechnol.* **40**, 282–287 (2018).

6. Mosbech, C., Holck, J., Meyer, A. & Agger, J. W. Enzyme kinetics of fungal glucuronoyl esterases on natural lignin-carbohydrate complexes. *Appl. Microbiol. Biotechnol.* **103**, 4065–4075 (2019).

7. Engh, R. A. & Huber, R. Accurate bond and angle parameters for X-ray protein structure refinement. *Acta Crystallogr. Sect. A* **47**, 392–400 (1991).
