# Supplementary material for: The structural basis of fungal glucuronoyl esterase activity on natural substrates
Source: Nat Commun. 2020 Feb 24;11:1026. doi: 10.1038/s41467-020-14833-9 (PMC7039992; doi:10.1038/s41467-020-14833-9)
Supplement: Supplementary file 2 — Peer Review [file 41467_2020_14833_MOESM2_ESM.pdf]

Reviewers' comments:

Reviewer #1 (Remarks to the Author):

Comprehension of glucuronoyl esterase activities is crucial for hydrolysis of lignin-carbohydrate complexes and efficient lignocellulosic biomass enzymatic degradation. The manuscript describes a set of fungal Cerrena unicolor GE crystallographic structures determined in the presence and absence of ligands. Both the full-length enzyme and its catalytic domain in active and inactive forms were studied. This structural analysis was further supported by SAXS, ITC and microscale thermophoresis experiments and shed light on molecular mechanisms of CE15-B recognition of LCC. This is a nicely written and comprehensive manuscript, which can be further improved by following suggestions below:

1. Line 90-91. The authors state that SAXS studies revealed that FL CuGE is a rigid two-domain molecule. How this is supported by SAXS data? Can the authors show correspondent Kratky plot(s) and discuss them?
2. Figure 2d-e show SAXS molecular envelopes of CuGE and demonstrate individual domains nicely fitted into two lobes of the SAXS-derived envelopes. However, the authors failed to show the linker. Could the authors model the linker and to discuss its conformation?
3. The authors show a putative monolignol docking site (Figure 6d), but do not demonstrate monolignol binding to this site. It would be instructive to model mono(di/poly)lignols bound to the putative binding site.
4. Supplementary Table 2: For the crystal structures determined in P4(1)2(1)2 space group one does not need to define neither crystallographic angles (always 90 deg) nor a and b parameters separately (a=b). Please, correct.

Reviewer #2 (Remarks to the Author):

The manuscript “The structural basis of fungal glucuronoyl esterase activity on natural substrates” by Ernst and colleagues is focused on structural studies of the family CA15 enzyme supported by microscale thermophoresis and isothermal calorimetry.
The task of separating chemically linked lignin and hemicellulose is one of the major obstacles preventing full utilization of the lignocellulosic biomass. The manuscript sheds new light, provides foundation for the structural understanding of the problem and adds valuable information to the body of knowledge in that area.
The manuscript is clearly written. The crystallography part is done at acceptable level with clean and correct modeling of the substrate binding sites. The structures might benefit from final ‘polishing’ to improve overall quality though. There are hundreds of very will visible strong unoccupied peaks in the electron density maps where water molecules should be placed (and some larger solvent molecules like glycerol and ethylene glycol), and some protein side chains are not modeled correctly (especially when alternative conformations are employed). More manual inspections and rebuilding of the models would be appreciated.
Few general remarks regarding the Supplementary table 2:
1) Listing 90 degrees alpha, beta and gamma is redundant, tetragonal space group cannot incorporate different numbers.
2) Formula should be provided for Rmerge in the footnote
3) What is I/s(I)? Is it avg(I) /avg(s(I)) or avg (I/s(I))?
4) Number of reflections belongs in the data collection part (with the number for the highest resolution shell included)
5) Rwork/Rfree should be reported in decimals, like the Rmerge earlier, not percents
6) Units of measure for B-factors are missing. Dots, not commas should be used in these lines.
7) RMSD bond lengths and angles are really RMS deviations FROM ideal values - this should be

indicated in the table - and the precision of the numbers should be made sensible (0.02 and 1.9, resp.) - also cite the source of the ideal values (e.g., Engh & Huber)
8) Include the Wilson B value in the table
9) Ramachandran statistics would be nice to have along with the note which program was used to calculate it.

Response to reviewer comments

Please find below our point-by-point response to reviewer comments. Replies to the reviewers' comments are indicated below in green text. Quotes from the manuscript are shown in *italics* with revisions shown; inserts underlined and ~~deleted sections with strike through~~.

In addition to the reviewer comments, the following additions to the text has been made in relation to Data Availability: L515 (Data availability section) now reads: *"The SAXS data and corresponding models presented in the manuscript has been deposited at SASBDB (www.sasbdb.org) with accession codes: SASDGD6 and SASDGC6. The crystal structures presented in this manuscript have been deposited in the Protein Data Bank with the PDB codes 6RTV, 6RU1, 6RU2, 6RV7, 6RV8 and 6RV9, respectively. It must be noted that the SAXS and the PDB models differ in their sequence numbering. The SAXS model use UNIPROT numbering whereas the PDB models refer to the sequence numbering illustrated in Fig 1.*
The source data underlying figures 1c, 5a, 5b, 5d and Supplementary materials Figure 1 are provided as a Source Data file."

Figure legends for Figure 1, Figure 5 and Supplementary Figure 1 now all includes a phrasing referring to the Source Data File.

Reviewer #1 (Remarks to the Author):

Comprehension of glucuronoyl esterase activities is crucial for hydrolysis of lignin-carbohydrate complexes and efficient lignocellulosic biomass enzymatic degradation. The manuscript describes a set of fungal Cerrena unicolor GE crystallographic structures determined in the presence and absence of ligands. Both the full-length enzyme and its catalytic domain in active and inactive forms were studied. This structural analysis was further supported by SAXS, ITC and microscale thermophoresis experiments and shed light on molecular mechanisms of CE15-B recognition of LCC. This is a nicely written and comprehensive manuscript, which can be further improved by following suggestions below:

1. Line 90-91. The authors state that SAXS studies revealed that FL CuGE is a rigid two-domain molecule. How this is supported by SAXS data? Can the authors show correspondent Kratky plot(s) and discuss them?

The dimensionless Kratky plots were and still are shown in Figure 2b. We recognize that additional comments and discussion are required and have inserted a couple of sentences that address the features of these plots.

L86 onwards now reads: "*Furthermore, the dimensionless Kratky plots shown in Fig. 2b for both samples, with their distinct bell-shaped curves reaching a plateau near zero at higher q values ~~(Fig 2b)~~, display the characteristics of rigid folded proteins. With a peak position in the dimensionless Kratky plot at $(sR_g)^2$= 1.1, the data for the dΔS270A variant indicate a compact and globular structure. The maximum interatomic distance (D_{max}) is 60 Å, derived from the pair distribution function P(r) (Fig. 2c).*
The SAXS data of the full length protein dWT, with an increased and right-shifted peak position in the dimensionless Kratky plot, display the characteristics of a multi domain elongated yet rigid particle."

2. Figure 2d-e show SAXS molecular envelopes of CuGE and demonstrate individual domains nicely fitted into two lobes of the SAXS-derived envelopes. However, the authors failed to show the linker. Could the authors model the linker and to discuss its conformation?

The low resolution structural information obtained from the SAXS data clearly shows rigidity of the structure and a fixed distance between the domains. However, given the nature of the data, there is some ambiguity in the orientation of the domains (as also stated in the manuscript (L106) and even more so in the linker conformation. Thus although models can be constructed with the linker, fitting the experimental data, this is not unambiguous and we have deliberately refrained from discussing the linker conformation to avoid over-interpretation of the SAXS data.

L97 has been revised to include a comment on this and now reads: *"These properties appear to be associated with the high content ~~the amino acids~~ of proline, threonine and serine in the linker region (Fig. 1c and Supplementary material Fig 2), which provides stiffness and potentially heavy O-glycosylation that may further reduce the flexibility of the linker[19]. Unfortunately it was not possible to unambiguously determine the conformation of the linker from the SAXS data."*

3. The authors show a putative monolignol docking site (Figure 6d), but do not demonstrate monolignol binding to this site. It would be instructive to model mono(di/poly)lignols bound to the putative binding site.

We acknowledge that the phrasing "docking site" could be misleading and that additional comments are required.

In contrast to the carbohydrate binding site, protein residues located at the "lignin-side" of the ester cleavage site are less conserved at the sequence level and strong specific interactions are not necessarily expected. However, the site is structurally conserved among fungal CE15-B enzymes. Conserved residues in this region that may interact with monolignol are H404, C269, C405 and S193. This site is not expected to be a high affinity binding site for monolignols in the absence of the carbohydrate-part of the substrate, but might serve as a binding site of the monolignol moiety once the interaction with the $XU^{m4}X$ portion of the substrate has been established. Therefore, we believe that it would be too speculative to model binding of monolignols at this point.

The text in the manuscript and the legend to Fig 6d have been revised to clarify this point.

L269 onwards now reads: "*In CuGE, this putative lignin binding surface is particularly rich in serine and threonine residues. In fungal CE15-B structures, a small depletion exists at the "lignin-side" of the ester cleavage site which is conserved at the structural level (Fig. 6d). Conserved residues that might interact with a monolignol moiety are S193, C269, H404 and C405."*
Figure legend for Fig 6d now reads: "***d)** Putative monolignol binding ~~docking~~ site."*

4. Supplementary Table 2: For the crystal structures determined in P4(1)2(1)2 space group one does not need to define neither crystallographic angles (always 90 deg) nor a and b parameters separately (a=b). Please, correct. This has been corrected.

A revised version of Supplementary Table 2 is provided. See further comments below.

Reviewer #2 (Remarks to the Author):

The manuscript “The structural basis of fungal glucuronoyl esterase activity on natural substrates” by Ernst and colleagues is focused on structural studies of the family CA15 enzyme supported by microscale thermophoresis and isothermal calorimetry.
The task of separating chemically linked lignin and hemicellulose is one of the major obstacles preventing full utilization of the lignocellulosic biomass. The manuscript sheds new light, provides foundation for the structural understanding of the problem and adds valuable information to the body of knowledge in that area.
The manuscript is clearly written. The crystallography part is done at acceptable level with clean and correct modeling of the substrate binding sites. The structures might benefit from final ‘polishing’ to improve overall quality though. There are hundreds of very will visible strong unoccupied peaks in the electron density maps where water molecules should be placed (and some larger solvent molecules like glycerol and ethylene glycol), and some protein side chains are not modeled correctly (especially when alternative conformations are employed). More manual inspections and rebuilding of the models would be appreciated.

The crystal structures are all determined to relatively high resolution (1.39-1.96 Å) and generally show very well-defined electron density. However, this also means that quite a large number of partially occupied molecules (water, glycerol, ethylene glycol as well as alternative protein side chain conformations) are visible in the electron density maps. Multiple cycles of manual inspection, rebuilding and refinement were performed and various strategies were tested to model the complex solvent regions. We decided on a conservative approach where only fully occupied solvent molecules were included. For complex solvent regions with overlapping components we have chosen to model only the predominant component (typically water OR glycerol). Furthermore, water molecules with B-factors > 80 $Å^2$, insufficient 2Fo-Fc density or close contacts to protein atoms (alternative conformations) were excluded. It is our impression that further manual inspection and rebuilding of the models will not significantly improve the quality of the structures.

The methods section, paragraph “Crystallization, data collection and structure determination”, L388 and onwards has been revised to include a comment on this: “*The data to high resolution and the complex composition of the solvent region comprised of water, glycerol, ethylene glycol molecules and the protein side chains in weakly populated alternative conformations made it non-trivial to model of the electron density in the solvent region. Only fully occupied solvent molecules were included in the final models, however a number of partial occupied solvent molecules are visible in the electron density maps.*”

Few general remarks regarding the Supplementary table 2:

A revised version of Supplementary table 2 is provided which accommodates the following comments.

1) Listing 90 degrees alpha, beta and gamma is redundant, tetragonal space group cannot incorporate different numbers.
This has been corrected.

2) Formula should be provided for Rmerge in the footnote

The formula for R_{merge} has been included in a footnote as requested.

3) What is I/s(I)? Is it avg(I) /avg(s(I)) or avg (I/s(I))?

We thank the reviewer for pointing out this ambiguity. It is the mean (I/s(I)). This has now been clarified in the table using the notation $\langle I/\sigma(I)\rangle$.

4) Number of reflections belongs in the data collection part (with the number for the highest resolution shell included)

As requested, we have now additionally included the number of unique reflections and number of reflections for the highest resolution shell in the data collection part.

5) Rwork/Rfree should be reported in decimals, like the Rmerge earlier, not percents

This has been corrected.

6) Units of measure for B-factors are missing. Dots, not commas should be used in these lines.

We thank the reviewer for noting this mistake. The unit for B-factors has been included ($Å^2$) and commas changed to dots as requested.

7) RMSD bond lengths and angles are really RMS deviations FROM ideal values - this should be indicated in the table - and the precision of the numbers should be made sensible (0.02 and 1.9, resp.) - also cite the source of the ideal values (e.g., Engh & Huber)

A footnote that clarifies this has been included in the table (with a reference to Engh & Huber1991) and the precision of the numbers has been corrected. Engh & Huber 1991 has been added to the reference list.

8) Include the Wilson B value in the table.

The Wilson B-factors have been added to the table.

9) Ramachandran statistics would be nice to have along with the note which program was used to calculate it.

As requested, the Ramachandran statistics have now also been added to the table.

The Methods section, paragraph "Crystallization, data collection and structure determination", from new L393 and onwards has been revised to include a reference to the program MolProbity and now reads: *"The Ramachandran statistics calculated by MolProbity[35] for the final refined models are as follows: 96.3 % in the favored region, 3.7 % allowed, 0 % outliers for apo-dΔS207A ….."*

Reference to Chen et al., 2010 has been added to the reference list as new reference 35.

REVIEWERS' COMMENTS:

Reviewer #1 (Remarks to the Author):

The authors responded all the raised questions in a satisfactory manner.

Reviewer #2 (Remarks to the Author):

I would like to thank the authors for answering all questions raised in the first stage of review and recommend the article to be accepted for the publication
